# Characteristic Features of Kynurenine Aminotransferase Allosterically Regulated by (Alpha)-Ketoglutarate in Cooperation with Kynurenine

**DOI:** 10.1371/journal.pone.0040307

**Published:** 2012-07-06

**Authors:** Ken Okada, Clement Angkawidjaja, Yuichi Koga, Kazufumi Takano, Shigenori Kanaya

**Affiliations:** 1 Department of Material and Life Science, Graduate School of Engineering, Osaka University, Osaka, Japan; 2 Core Research for Evolutional Science and Technology, Japan Science and Technology Agency, Osaka, Japan; Institute of Enzymology of the Hungarian Academy of Science, Hungary

## Abstract

Kynurenine aminotransferase from *Pyrococcus horikoshii* OT3 (PhKAT), which is a homodimeric protein, catalyzes the conversion of kynurenine (KYN) to kynurenic acid (KYNA). We analyzed the transaminase reaction mechanisms of this protein with pyridoxal-5′-phosphate (PLP), KYN and α-ketoglutaric acid (2OG) or oxaloacetic acid (OXA). 2OG significantly inhibited KAT activities in kinetic analyses, suggesting that a KYNA biosynthesis is allosterically regulated by 2OG. Its inhibitions evidently were unlocked by KYN. 2OG and KYN functioned as an inhibitor and activator in response to changes in the concentrations of KYN and 2OG, respectively. The affinities of one subunit for PLP or 2OG were different from that of the other subunit, as confirmed by spectrophotometry and isothermal titration calorimetry, suggesting that the difference of affinities between subunits might play a role in regulations of the KAT reaction. Moreover, we identified two active and allosteric sites in the crystal structure of PhKAT-2OG complexes. The crystal structure of PhKAT in complex with four 2OGs demonstrates that two 2OGs in allosteric sites are effector molecules which inhibit the KYNA productions. Thus, the combined data lead to the conclusion that PhKAT probably is regulated by allosteric control machineries, with 2OG as the allosteric inhibitor.

## Introduction

Kynurenine-oxoglutarate transaminase (EC 2.6.1.7), also known as kynurenine (KYN) aminotransferase (KAT) [1], is a dimeric enzyme containing 2 covalently-bound pyridoxal-5′-phosphates (PLP) as cofactor moieties and is the last enzyme in the kynurenic acid (KYNA) biosynthetic pathway. Kynurenic acid (KYNA) is biosynthesized as a product of the normal metabolism of the amino acid l-tryptophan and via a KYN intermediate; KYNA is synthesized via the transamination of KYN in the presence of KAT. KYNA is sequentially biosynthesized from l-KYN via a 4-(2-aminophenyl)-2,4-dioxobutanoate (4AD) intermediate by KAT (Fig. S1). KAT transfers the amino group of KYN to α-ketoglutaric acid (2OG) via pyridoxamine phosphate (PMP), thus synthesizing l-glutamic acid (Glu).

In human brain, KYNA acts as a natural antagonist of the glycine site of NMDA (*N*-methyl d-aspartate) receptor (NMDAR) and plays a key role in the glutamatergic neurotransmission system [2]. It is also thought to be involved in the pathogenesis of diseases such as Alzheimer’s [3] and schizophrenia [4]. Recently, genes encoding KATs were isolated from various organisms, including human [5], mouse [6], *Aedes* [7], *Saccharomyces* [8], and a protozoan parasite of *Trypanosoma* that causes fatal sleeping sickness in human [9]. The KAT from the hyperthermophilic archaeon, *Pyrococcus horikoshii* OT3 (PhKAT), is a homolog of human KAT II (HuKAT II) and exhibits low but significant amino acid sequence homology with the KATs of various organisms.

The crystal structure of the apo-form of PhKAT has been solved at 2.2 Å resolution [10]. Although this protein was previously designated PhKAT-II, it is simply designated PhKAT in this study because other homologs that are highly conserved relative to PhKAT are absent from the genome. In *P. horikoshii* OT3, although the genes that are involved in the biosynthesis of KYN from l-tryptophan are unidentifiable in the genome, PhKAT that catalyzes the formation of KYNA and a human kynureninase homolog related to the biosynthetic pathway from KYN to NAD are present in the genome [11], [12]. This suggests that the KYNA pathway is probably present in *P. horikoshii*. However, the physiological functions of KYNA in this hyperthermophilic archaeon remain unclear. Therefore, we characterized the KYNA biosynthesis abilities of PhKAT to clarify its biochemical function.

To understand the action mechanisms of PhKAT and compare them with those of their counterparts in humans, other mammals, yeasts, *Aedes*, and protozoan parasites, we characterized the reactions catalyzed by this enzyme. We also analyzed the interaction between PhKAT, PLP cofactor, and 2OG substrate by using spectrophotometric technique and isothermal titration calorimetry (ITC). ITC results indicate that PhKAT and PLP and/or 2OG interact with a high affinity. The enzyme kinetic analyses revealed that PhKAT is an allosteric enzyme and that transaminated acceptors for PhKAT are 2OG and oxaloacetic acid (OXA). Furthermore, in the crystal structure of PhKAT complexed with an allosteric effector, 2OG was bound to PhKAT at a rate of 4 molecules per protein. Two 2OG molecules were identified in the binding pocket of PhKAT other than a pocket constructed for active sites. This demonstrates that 2OG is an allosteric effector necessary for the regulation of KYNA biosynthesis.

## Results

### Expression and Purification of PhKAT

**Figure 1:**
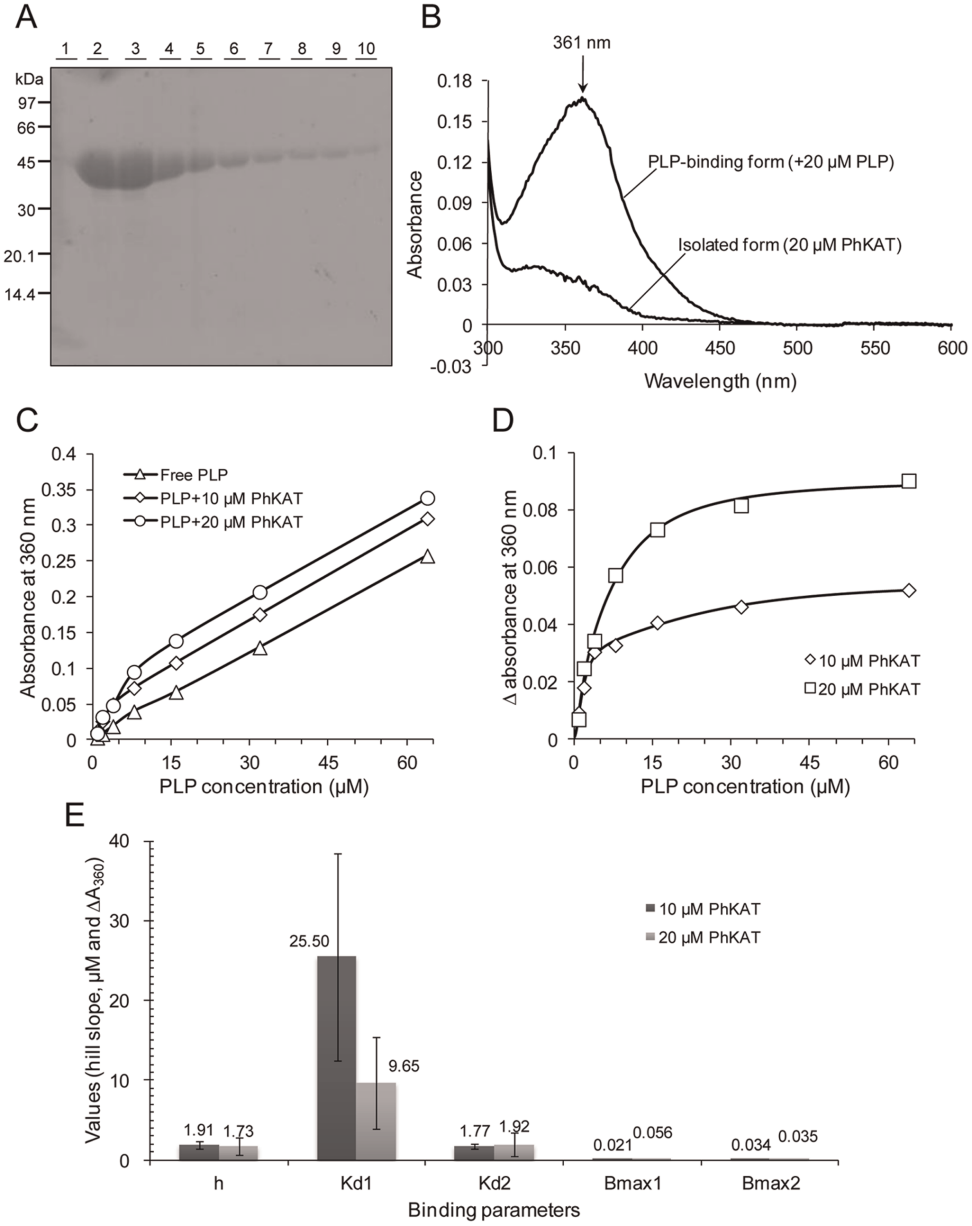
One-step purification of PhKAT and spectrophotometric analysis of the cofactor (PLP) binding to apo-KAT. (A) SDS-polyacrylamide gel electrophoresis (SDS-PAGE). Lanes ∼1–10, verification of the purity of PhKAT in each elution fraction from the metal-affinity column. Protein fractions in lanes ∼2–10 were used for further analysis and crystallization. The positions of the protein standards (molecular masses, 97, 66, 45, 30, 20.1, and 14.4 kDa) are indicated. (B) UV–visible absorption spectra of as-isolated PhKAT (lower spectrum) and its cofactor-binding form (holo-PhKAT, upper spectrum). The lower spectrum was obtained with the as-purified protein, and the upper spectrum was recorded after mixing 20 µM PhKAT with 20 µM PLP. The spectrum shows a maximum at 361 nm. (C) Spectrophotometric titration of PLP for 10 and 20 µM PhKAT. (D) Relationship between the equilibrium-binding response and the concentrations of PLP and PhKAT. The solid lines represent the fitting curve obtained by the 2-site binding model with hill slopes using Prism5 software. (E) Summary table of binding parameters from D. Errors are the S.E. from the fit of the data. The binding affinity of a second binding site for PLP was stronger than a first binding site in PhKAT (*K*_d_1>*K*_d_2).

*E. coli* BL21-CodonPlus (DE3) cells were transformed with pET28a-PhKAT. PhKAT expression was induced with IPTG. PhKAT (48 kDa), which was expressed as a soluble protein, was detected by sodium dodecyl sulfate (SDS)-PAGE (Fig. 1A). Recombinant PhKAT was purified using a His TALON cartridge at the first step; it was identified as single 51-kDa bands (Fig. 1A, lanes 2–10). A 2-L culture yielded 17 mg purified PhKAT. The absolute activities of PhKAT were maintained even after 12 months of storage (−80°C) in 50 mM HEPES–NaOH buffer (pH 7.5) containing 100 mM NaCl.

### PLP Binding to PhKAT

A characteristic of KAT is its ability to form a Schiff-base link with the PLP cofactor (PLP complex). To determine whether PhKAT can form such a complex, it was incubated with PLP, and the changes in the absorption spectrum were recorded. The change in the absorption spectrum upon the formation of the PLP–PhKAT complex is depicted in Figure 1B.,The maximum peak of PLP-PhKAT complex was observed at 361 nm, blue-shifted from the that of free-PLP at 390 nm. Futhermore, the free-PLP peak at 330 nm disappeared (Fig. S2). Figure 1C and D illustrates the titration plots obtained for PhKAT and clearly indicates that PhKAT binds with PLP. The titration of PhKAT with PLP was followed by the observation of the absorbance changes at 360 nm (Fig. S2). The binding points of PhKAT with PLP were unable to be fitted using the 1-site binding model. Therefore, they were fitted using the 2-sites binding model with hill slopes (Equation S1), as determined by ITC. The dissociation constants for PLP in the 10 and 20 µM PhKAT conditions were *K*_d_1 = 25.5 and *K*_d_2 = 1.77, and *K*_d_1 = 9.65 and *K*_d_2 = 1.92 µM, respectively (Fig. 1E). The results indicate a positive cooperativity of PLP binding.

### Comparison of the Crystal Structures of the PLP–PhKAT Complex and Human KAT II (HuKAT II)

Crystals of PLP-bound PhKAT belong to space group C2 and have unit cell parameters a, b, and c of 85.927, 71.055, and 136.348 Å, respectively (Table S1). The refined model of the PLP–PhKAT complex contains 2 PhKAT molecules (homodimeric), 404 of 428 residues (residues 25–428), 2 PLPs, and 585 water molecules in the asymmetric unit. The electron density of residues 1–24 and the N-terminal His-tag was not observed probably due to structural disorder. The final crystallographic R-factors and free R-factors with isotropic temperature factors are 18.4% and 23.2%, respectively, for 82,018 unique reflections in the resolution range of 50–1.72 Å. Data collection and refinement statistics are summarized in Table S1.

**Figure 2:**
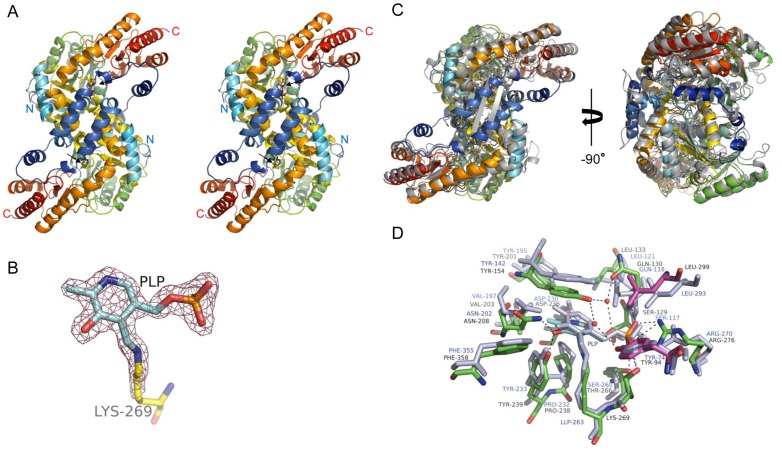
Overall crystal structure of the PLP–KAT complex and PLP in complex. (A) Stereoview and cartoon representation of the structure of functional PhKAT dimer bound to 2 PLP cofactors. A C-α trace is shown with rainbow coloring from N- (blue) to C-termini (red). Black arrows indicate the position of PLP molecules and active sites. (B) Part of PLP with a 2F_o_-F_c_ electron density map contoured at 1.5 σ. PLP molecules formed a Schiff-base link with lysine-269. (C), (D) Superimposed representation of the structures of PhKAT and HuKAT II. (C) Cartoon representations of PLP complex structures of PhKAT (PDB code: 3AOV) and HuKAT II (PDB code: 2VGZ) after optimal superimposition. PhKAT and HuKAT II are rainbow-colored and gray, respectively. The left-hand molecule is shown in the same orientation as in A, whereas the right-hand molecule is rotated −90°. (D) Stick representation of the PLP cofactor-binding sites with PhKAT and HuKAT II after optimal superposition of the 2 structures. HuKAT II is colored blue-gray. These results indicate that the secondary structures of KAT are conserved between *P*. *horikoshii* and humans. The figure was generated using PyMOL.

Two peptide chains (designated chains A and C) were located in an asymmetric unit and form a functional homodimer (Fig. 2A). Two PLP cofactors formed a Schiff-base link with Lys269 (Fig. 2B). The results indicate that the conformation of the PLP–PhKAT complex is functionally active (Fig. S3). The overall superimposition of the main chain structures of PhKAT and HuKAT II are shown as cartoon drawings in Figure 2C. The structures are superimposed along the C-α atoms of residues 25–428 and the 2 PLP cofactors in PhKAT, and the C-α atoms of residues 1–18 and 32–425 and the PLP cofactors in HuKAT II [13], [14]; this is because HuKAT II lacks residues 19–31 compared with PhKAT. The root mean square deviation value for the C-α atom between the 2 structures calculated by PyMOL is 1.8 Å. The overall secondary structures of PhKAT were fairly similar to those of HuKAT II [14] and also resembled the other known backbone structures of KAT families. However, it should be noted that the backbone conformation of the α-helix of Glu73–Glu86 in PhKAT is significantly different from the corresponding conformation of HuKAT II, which is a β-sheet as shown in the superimposition (Fig. 2C). The active site contains 1 PLP molecule covalently linked to Lys269 and is located in a deep cleft at the domain interface built up by residues from both subunits (Fig. 2D). PLP-bound pockets are conserved between PhKAT and HuKAT II (Fig. 2D). The constituted residues of the active sites around PLP-bound pockets are all conserved, except for Ser260 in HuKAT II (Fig. 2D). The amino acid sequences of PhKAT and HuKAT II share low homology (Fig. S4); the homology with HuKAT II is as low at 27%, but the cofactor-binding site and PLP-ligand lysine that form a Schiff-base linkage in PhKAT are fairly conserved as shown in their sequence alignment (Fig. S4).

### Spectrophotometric Evidence of KAT Activity and KYNA Synthesis

**Figure 3:**
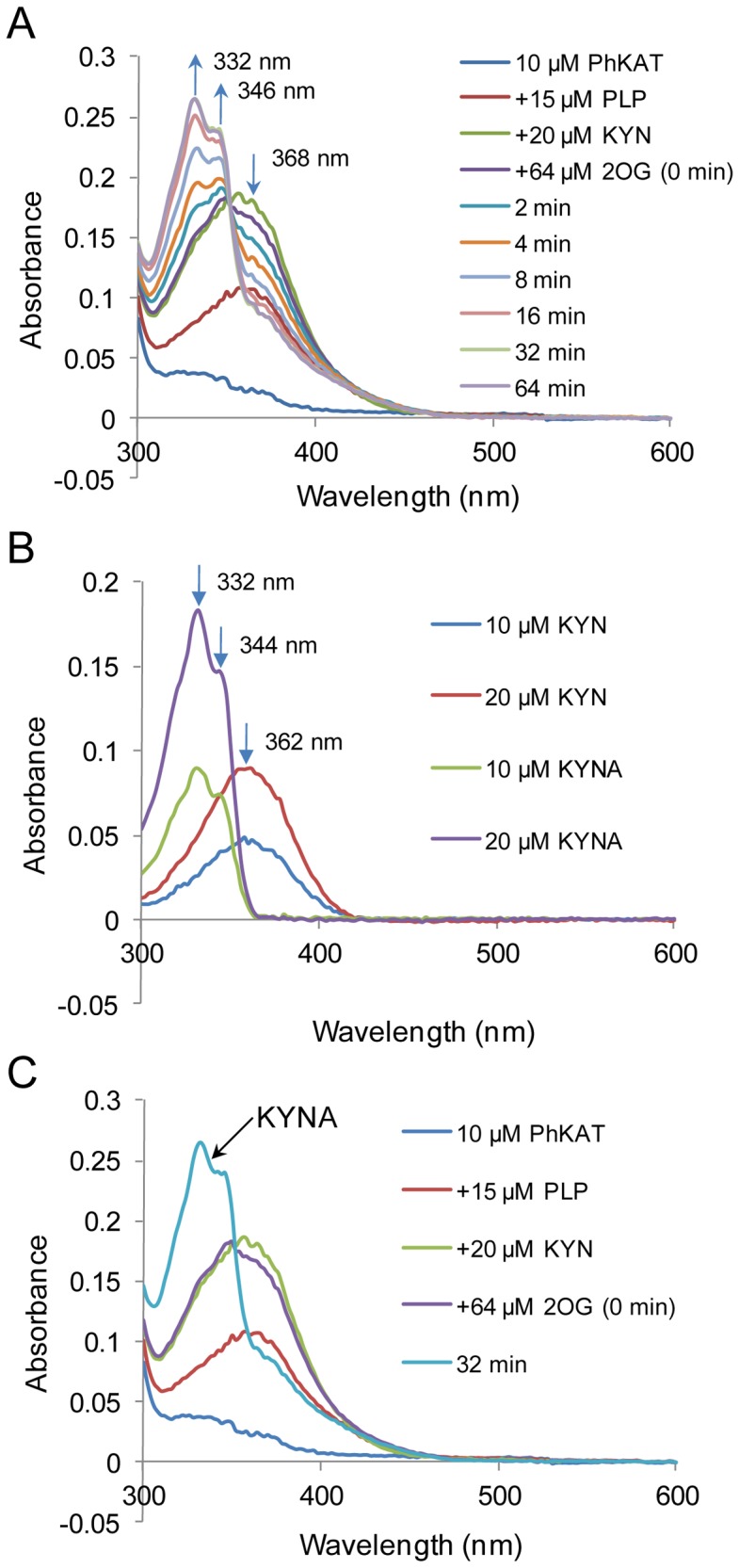
Catalytic activity of PhKAT. (A) Spectrophotometric assay of the time course of the PhKAT-catalyzed activity of KYN. The PhKAT-catalyzed activity of KYN was examined by spectrophotometry using both PLP and 2OG. Time-dependent absorbance changes were monitored during the PhKAT reaction after the addition of 2OG. The arrows indicate the direction of absorbance changes during incubation. The absorption band at 368 nm decreases with time, while bands at 332 and 346 nm appear and increase, respectively. The spectrum at 32 min was identical to that at 64 min. (B) Comparison of the absorption spectra of KYN and KYNA. The absorption spectra of KYN and KYNA measured at concentrations of 10 and 20 µM. The spectrum of KYNA exhibits 2 peaks at 332 and 344 nm, and KYN exhibits a peak at 362 nm. (C) The observed absorbance before and after the PhKAT catalyzed-reaction. The final product of the PhKAT-catalyzed reaction from KYN substrate was identified with KYNA.

Previously, we confirmed the conversion of KYN to KYNA by HPLC [15]. However, whether the conversion to KYNA is an enzymatic reaction remains unknown. Thus, we monitored the PhKAT-catalyzed reaction spectrophotometrically (Fig. 3). When PhKAT, PLP, and KYN were incubated with 2OG, the KYN peak present at 368 nm disappeared and 2 peaks with maxima at 332 and 346 nm appeared in a time-dependent manner (Fig. 3A). Spectrum changes at 332 and 346 nm were saturated at 32 min after the start of the reaction. KYNA exhibited maximum peaks at 332 and 344 nm (Fig. 3B). Thus, the 2 peaks at 332 and 346 nm in Figure 3A indicate the presence of KYNA. The disappearance of the peak at 368 nm in Figure 3A shows that KYN is enzymatically converted to KYNA as observed when comparing spectrum changes (Fig. 3C). This result indicates that PhKAT from this hyperthermophilic archaeon is a KAT.

### Kinetics of the Transamination Reaction from KYN to 2OG by KAT

**Figure 4:**
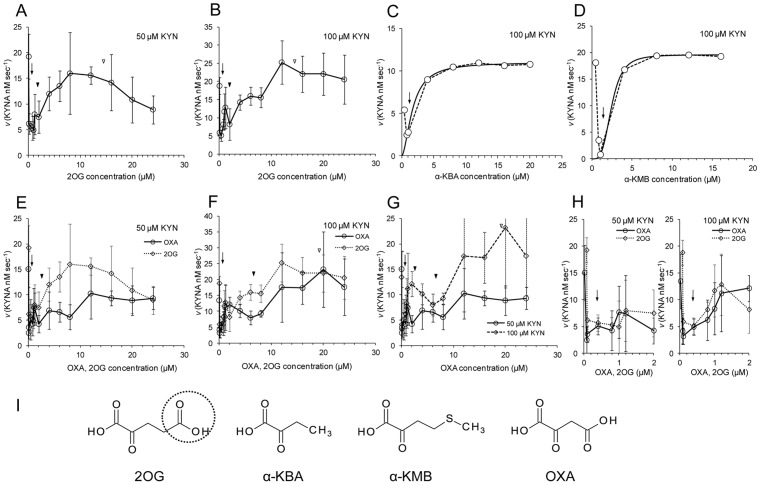
Kinetics of the KAT reaction with 2 substrates. The KAT-catalyzed reaction from KYN to KYNA was determined by monitoring the change in absorption at 332 nm. (A), (B) Measurement of the KAT-catalyzed conversion to KYNA with definite KYN concentrations, and the allostery of 2OG for PhKAT. Values are mean±S.D. ((A), (B): n = 5). The arrow and arrow heads indicate the conformation changes of PhKAT from R state to T state in conjunction with binding of 2OG to a first binding site and allosteric inhibitions, respectively. The KYNA productions from KYN by KAT are regulated at two respects of low and high 2OG concentrations. Black arrow-head: first allosteric inhibition by a first 2OG effector molecule; white arrow-head: second allosteric inhibition by a second 2OG effector molecule. (C), (D) The PhKAT-catalyzed reaction by α-keto acid analogs. The solid line represents the fitting curve obtained by allosteric sigmoidal model using Prism5 software (Equation S3). The arrows indicate the conformation changes from R state to T state in conjunction with binding of keto acid analogs to a first binding site. (E), (F) Comparison of the PhKAT-catalyzed reaction by OXA and 2OG, and the allostery of OXA for PhKAT. Values are mean±S.D. ((E), (F): n = 5 and 4, respectively). The arrow indicates the conformation changes of PhKAT from R state to T state in conjunction with binding of OXA to a first binding site. Black arrow-heads: first allosteric inhibition by OXA; white arrow-head: binding of fourth KYN. (G) Comparison of E and F. (H) Magnified views of E and F (∼2 µM). (A), (E) 50 µM KYN; (B), (C), (D), (F) 100 µM KYN. (I) Keto acids: 2OG, α-KBA, α-KMB and OXA. A dot circle indicates the important parts in 2OG for the allostery.

The transamination abilities of PhKAT from KYN to 2OG, which were used to monitor KYNA production, were investigated by spectrophotometry. In this reaction, KYN is converted to KYNA via transamination from KYN to 2OG via PLP in the presence of excess KYN. The conversion velocities of KYN to KYNA are plotted with respect to 2OG concentrations as the enzymatic abilities of PhKAT at pH 7.5 (Fig. 4). The reaction curve of PhKAT indicates that it is allosteric sigmoidal and allosteries (Fig. 4A and B) and that the catalytic activity of KAT is strongly inhibited by high 2OG concentrations (two times of that of KAT) (Fig. 4A and B). This result indicates that 2OG probably is an allosteric inhibitor. In addition, the KYNA productions from KYN by KAT were regulated at two respects of the low and high 2OG concentration regions. The result indicates that 2OG as allosteric inhibitor functions at 2 molecules for PhKAT, and affinities between two subunits of PhKAT formed homo-dimer for first and second 2OG effectors differ. The absolute inhibition constant (*K*_i_) was 20.11 µM (Fig. S5A, Equation S2 and Table S2). The results demonstrate that 2OG as allosteric inhibitor binds at a rate of 2 molecules per PhKAT as the homo-dimer. The velocities of KYNA synthesis by PhKAT at first contact of 2OG substrate (low concentration) accelerated to nearly “*V*_max_” (Fig. 4A and B). This behavior indicates that the conformation of PhKAT changes from R state (high affinity) to T state (low affinity) in conjunction with that 2OG binds to a first binding site of PhKAT (Fig. S5B). In addition, the slope of the velocity curves at low 2OG concentration region (∼1.2 µM) in Figure 4B (Fig. S5B right hand) sifted to low 2OG concentrations more than in Figure 4A (Fig. S5B left hand). This result indicates that KYN functions as an activator for KYNA production (Fig. S5B). In fact, the acceleration and recovery of KYNA synthesized velocities were observed in Figure 4A and B. The results indicate that KYN as an activator probably unlocks the inhibiting action of 2OG for PhKAT. The α-KBA and α-KMB keto-acid analogs were able to support the KAT reaction. H-values were greater than 1 (Table S2 and Equation S3). H-values for α-KBA, and α-KMB were 1.79 and 3.57 under 100 µM KYN, respectively. The results show that PhKAT is an allosteric enzyme characterized by positive cooperativity and ligands for PhKAT are 2 and 4 molecules, respectively. However, α-KMB does not bind with PhKAT at 4 molecules due to that a molar ratio discords between PhKAT and α-KMB. It might indicate that *K*_m_s of α-KMB change by that 2 KYN bind with PhKAT. Therefore, Figure 4C shows only the two reactions by α-KBA as substrate without an allosteric inhibition and binding with KYNs as an activator. *K*_m_ values for α-KBA, and α-KMB were 1.73 and 2.41 µM under 100 µM KYN, respectively (Table S2). The velocities of KYNA synthesis by PhKAT at first contact of α-KMB accelerated to around “*V*_max_” (Fig. 4D). The result indicates the conformation changes of PhKAT from R state to T state in conjunction with binding of α-KMB to a first binding site of PhKAT. The velocity changes at 0.4 µM in α-KBA are smaller than α-KMB (Fig. 4C). This might indicate that PhKAT is the R’ state which is an intermediate position between R state and T state. Thus, KYN as an activator might be able to bind with PhKAT when is T state. OXA was able to support the KAT reaction and allostery including allosteric inhibition (Fig. 4E, F and G). However, the allosteric inhibitions were cause by only binding of OXA with a first allosteric site (Fig. 4E and F). This result supports that PhKAT has been regulated by 2OG. *V*_max_s supported by OXA were lower than 2OG (Fig. 4E and F). These results may indicate that a KYNA synthesis has been driven by 2OG. Although results showed the binding of third and fourth KYNs to PhKAT (Fig. 4E and F), the recovery from an inhibition by an OXA which binds with one allosteric site was not observed at 100 µM KYN condition (Fig. 4F). Additionally, figure 4G indicates that KYN activates PhKAT, as with 2OG (Fig. 4B). The results indicated the conformation changes of PhKAT from R state to T state in conjunction with binding of OXA to the first binding site of PhKAT (Fig. 4H). The affinity for OXA at 1 µM was higher than 2OG in 50 µM KYN conditions (Fig. 4H left hand) and that was lower than 2OG in 100 µM KYN conditions (Fig. 4H right hand). Affinities for 2OG at low concentrations (∼0.08 µM) were higher than OXA (Fig. 4H). Affinities for OXA at 0.02 µM concentrations were a 50 µM KYN condition higher than a 100 µM KYN condition (Fig. 4G). These indicate that PhKAT changes the nature of transaminated acceptors in conjunction with changes in concentration of KYN and that a KYNA biosynthesis is driven by 2OG and/or OXA at low concentration. The combined results suggest that the transaminase reaction of PhKAT is specifically regulated by 2OG as an allosteric effector, and that a carboxyl group of C5 in 2OG performs the important role for allosteric inhibitions. Furthermore, the length of keto acids probably relate to the cooperation with KYN as an activator and binding to two allosteric sites (Fig. 4I).

### Thermodynamics of PhKAT, its Cofactor, and Substrate Interactions

**Figure 5:**
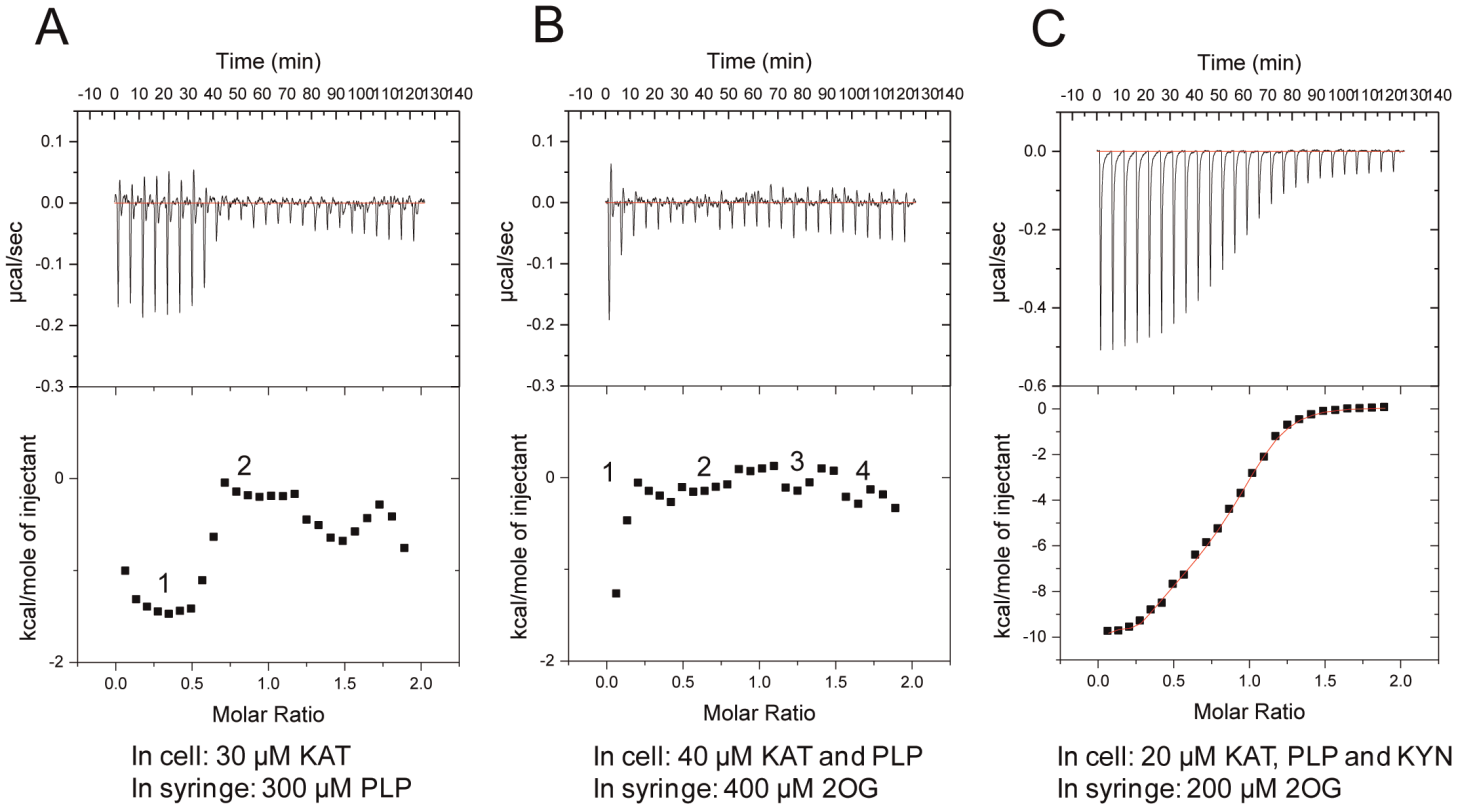
ITC analysis of the interaction between the cofactor, substrates, and KAT. The ITC profiles include experimental conditions. (A) KAT and PLP; (B) KAT-PLP and 2OG; (C) KAT-PLP-KYN and 2OG. The numbers indicate a binding site. The binding between the KAT–PLP complex and 2OG might be an overestimation of the limit of ITC. (A), (B) The shapes of binding curves indicated that the dissociation constants for PLP (as apparent constant) and 2OG of second and first binding sites, respectively, might be approximately femtomole orders. (C) A curve fitting was performed by using a sequential binding 4-site model.

ITC was used to accurately determine the binding parameters of purified PhKAT, PLP and/or KYN, and 2OG. Figure 1A shows the protein purity used in these experiments, and Figure 5 and S6 show representative ITC experiments including the titration data and binding curves calculated using the best-fit parameters. Table S3 summarizes the thermodynamic parameters. The ligands bind to PhKAT in 1∶1 and 1∶2 ratios. The PLP cofactor binds to 2 sites of PhKAT (Fig. 5A), indicating that it binds to 2 active sites of functional homodimeric PhKAT. The 2 sites where PLP binds to PhKAT were observed in the crystal structure of the PhKAT–PLP complex at 1.72 Å (Fig. 2). A shape of binding curves indicated that the dissociation constant for PLP (as apparent constant) of a second binding site in PhKAT might be an approximately femtomole order. A 2OG substrate binds to 4 sites of the PhKAT–PLP complex (Fig. 5B). However, the binding of 2OG to the PhKAT–PLP complex possibly is an overestimation of the limit of ITC. A shape of binding curves indicated that the dissociation constant for 2OG of a first binding site in PhKAT might be an approximately femtomole order. The 4-sites binding of 2OG indicate that two 2OGs may operate as an allosteric effector molecule that regulates the transaminase reaction. In fact, the transaminase activity of PhKAT at steady-state kinetic analysis was inhibited at 2 respects of low and high 2OG concentrations (Fig. 4A and B). These results demonstrate that it binds to 4 binding sites, which are the 2 active and allosteric sites, of functional homodimeric PhKAT. A 2OG substrate binds to 4 sites of the PhKAT–PLP–KYN complex with dissociation constants (*K*_d_s) of *K*_d_1 = 238.15 nM and *K*_d_2 = 15.80 µM (as substraes) and *K*_d_3 = 3.12 µM and *K*_d_4 = 1.09 µM (as allosteric effectors), and the negative and positive cooperativities, respectively (Fig. 5C). 2OG binds to PhKAT more weakly in the presence of KYN than in its absence (Fig. 5B). Although Figure 5C is fitted by a 4-sites binding model, this binding curve fits for use other binding models in a similar manner. Therefore, the binding numbers might need to be determined by using other methods. This study determined the binding numbers of 2OG for PhKAT from Figure 5B, enzyme kinetics and crystal structure analysis. When the affinities of 2OG for PhKAT decreases and/or increase, it cooperates with KYN and/or 2OG in conjunction with binding to each sites. The stoichiometry between PhKAT-PLP complex and 2OG might get out of order due to the binding gears between binding sites in PhKAT (Fig. 5B and C). The PhKAT-PLP complex precipitated when mixed with 2OG (data not shown). The binding powers of 2OG may be too much for PhKAT. Therefore, the molar ratios between its complex and 2OG might be out of alignment. Additionally, in enzyme kinetic analyses, the affinities of PhKAT for 2OG varied in response to changes in concentrations of KYN. Therefore, ITC experiments might need to be done with modified KYN concentrations in the cell.

### PhKAT, PLP, and 2OG Complex Structures and Their Electrostatic Surface Potential

To examine the binding mechanisms of PhKAT and 2OG, the crystallized PhKAT–PLP–2OG complex was formed in a 1∶1:1 ratio. Crystals of the PhKAT–PLP–2OG complex belong to space group C2 and have unit cell parameters a, b, and c of 85.774, 70.977, and 136.723 Å, respectively (Table S1). The refined model of the 2OG–PLP–KAT complex contains 2 KAT molecules (homodimeric), 404 of 428 residues, 2 PLPs, 2 2OGs, and 838 water molecules in the asymmetric unit. No electron density for residues 1–24 was observed probably due to structural disorder. The final crystallographic R-factors and free R-factors with isotropic temperature factors are 17.7% and 22.4% for 109,393 unique reflections in the resolution range of 50–1.56 Å. Refinement statistics are summarized in Table S1.

**Figure 6:**
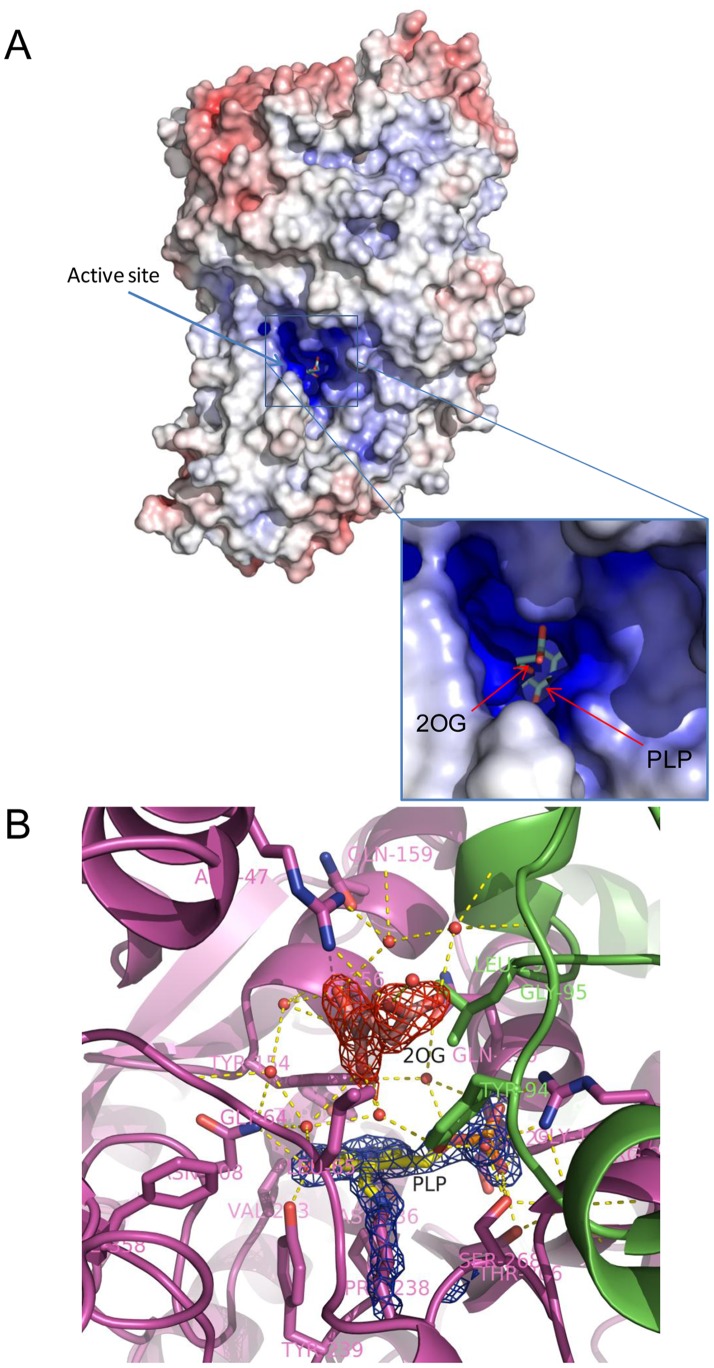
Surface and substrate binding site representations of 2OG, PLP, and KAT complexes. (A) Electrostatic potential mapped onto the molecular surface of a functional KAT dimer with bound 2OG and PLP shown as sticks. The 2OG/PLP-bound site of KAT is positively charged. Electrostatic potential calculated using APBS and PDB2PQR server. Potentials are contoured from −15 kT/e (negative charge, red) to +15 kT/e (positive charge, blue). The positively charged sites bind to both the substrates and cofactor with high affinity. (B) Close-up view of a 2OG-bound site of KAT structure. Parts of 2OG and PLP with 2F_o_-F_c_ electron density maps contoured at 1.5 σ. The figure was generated using PyMOL.

Electrostatic potentials can induce attractive forces and have the longest range of any chemical interaction; in turn, short-range forces become increasingly important. In an effort to identify structural features in the PhKAT protein that could promote interactions with a 2OG substrate, we assessed whether electrostatic complementarities are possible. Thus, we determined the crystal structures (at 1.56 Å resolution) of 2OG, PLP, and the PhKAT triple complex at the binding site to the PhKAT subunit with one 2OG substrate. Figure 6 shows the electrostatic surface potentials and locations of constituted residues of the active sites around 2OG- and PLP-bound pockets of the PhKAT–PLP–2OG triple complex structure. A single 2OG was bound to a positively charged active site (Fig. 6A). These results indicate that the interaction between the substrate and cofactor with PhKAT is electrostatic binding, which capacitates the binding between 2OG and PLP with PhKAT with high specificity and affinity.

### Overall Structure of PhKAT Complexed with 2OG (3ATH), and Comparison with Other PhKAT Complex Structures

The crystals of 2OG-bound PhKAT belong to space group C2 and have unit cell parameters a, b, and c of 85.817, 70.989, and 136.816 Å, respectively (Table S1). The refined model of PhKAT in complex with 2OG contains 2 PhKAT molecules (homodimer), 404 of 428 residues, 2 PLPs, 4 2OGs, and 628 water molecules in the asymmetric unit. No electron density for residues 1–24 was observed probably due to structural disorder. The final crystallographic R-factors and free R-factors with isotropic temperature factors are 17.0% and 21.5%, respectively, for 86,699 unique reflections in the resolution range of 50–1.69 Å. Refinement statistics are summarized in Table S1.

**Figure 7:**
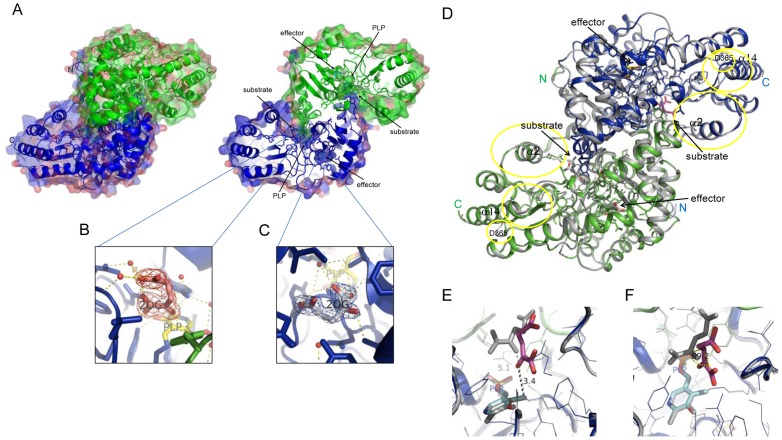
Overall crystal structure of PhKAT in complex with PLP and 2OG as a substrate and allosteric effector (PDB code: 3ATH). (A) Surface and cartoon representation of the structure of functional PhKAT dimer bound with 2 PLP cofactors and 4 2OGs. A Cα trace is shown with blue and green coloring with 2 subunit chains. Black arrows indicate the position of PLP as cofactor and 2OG as substrate and allosteric effector. Left hand, cartoon-and-surface representation of the allosteric–effector complex. Right hand, cut-away of the overall structure of the left-hand complex. (B), (C) Part of 2OG as a substrate (B) and as an allosteric effector (C) with a 2F_o_-F_c_ electron density map contoured at 1.3 σ. (D), (E), (F) Superimposed representation of 2OG complex structures of PhKAT. (D) Cartoon representations of 2OG complex structures of PhKAT after optimal superimposition. 2OG as a substrate complex (PDB code: 3AOW) and 2OG as a substrate and an allosteric effector complex (PDB code: 3ATH). Gray cartoon-and-line represents PhKAT bound only to 2OG as a substrate complex; PhKAT bound to 2OG as substrates and allosteric effectors is colored green and blue for 2 subunits. Yellow circles show regions with significant conformation changes. Black arrows indicate the position of 2OG as substrates and allosteric effectors. (E), (F) Stick-and-line representation of the PLP-cofactor binding sites and 2OG as substrate after optimal superposition of the 2 PhKAT complex structures. 2OG as a substrate complex only is colored gray. (E) Black and gray labels, and dotted lines show the distance (Å) between 2OG and the C4A atom of PLP in a Schiff-base link with lysine-269. (F) Angles (°) of 2OG between substrate-bound PhKAT, and substrate- and allosteric effector-bound PhKAT structures. The figure was generated using PyMOL.

Two peptide chains were located in an asymmetric unit and form a functional homodimer (Fig. 7A, left molecule). Two PLP cofactors formed a Schiff-base link with Lys269, and 4 2OG molecules were bound to 2 binding pockets within the active sites or bound to pockets close to the lysine Schiff-based PLP. Surprisingly, the latter pockets are located on the back of the active sites of PhKAT (Fig. 7). The results indicate that 2OG as a substrate is an allosteric effector of PhKAT.

The overall structure of the superimposition of the main chain structures of PhKAT–2OG complexes 3AOW and 3ATH, which are 2 different structures with 2 and 4 2OGs as substrates, respectively, with the 2OG-bound PhKAT are shown as cartoon drawings in Figure 7D. The structures are superimposed along the C-α atoms of residues 25–428. Furthermore, there are 2 PLP cofactors in PhKAT complexed with 2 2OGs as substrates, and the C-α atoms of residues 25–428 and the 2 PLP cofactors in PhKAT are complexed with 4 2OGs as substrates and allosteric effectors. Although the overall secondary structure of PhKAT complexed with 2 2OGs as substrates was similar to that of PhKAT complexed with 4 2OGs as substrates and allosteric effectors, the areas around the α2 and α14 helices, and the D-365 region had significantly differently conformational changes (Fig. 7D). The coassemblies of 2OG as an allosteric effector of PhKAT in complex with 2OG as substrate caused a major change in the spatial layout of 2OG as a substrate (Fig. 7E and F). The distances between the C4A atoms of PLP and 2OG when the 2OGs were in a substrate-bound and a substrate and allosteric effector-bound form were 5.1 and 3.4 Å, respectively (Fig. 7E). As part of an allosteric effector complex, 2OG was altered at 89.2° (Fig. 7F). The results indicate that the binding of 2OG to allosteric sites directly affects substrates in the active sites of PhKAT. This indicates that the substrate binding force undergoes a change due to the binding of 2OG to allosteric sites. The inhibition of KAT activity by allosteric effectors might be because KYN is prevented from approaching PLP reaction sites by 2OG as substrate (Fig. 7E and F). Furthermore, the binding of 2OG to allosteric sites induced a conformation change of the α2 helix constructed active sites of PhKAT (Fig. 7D and Fig. S7). The distance between the Cα-traces of a PLP complex and an allosteric effector complex was a maximum of 1.3 Å (Fig. S7). The results indicate that the binding of 2OG to allosteric sites induces an imperceptible effect on the conformation of the 2 active sites of PhKAT.

### Evolutionary Conservation of Allosteric Effector-binding Sites Between PhKAT and Mammalian KAT II

**Figure 8:**
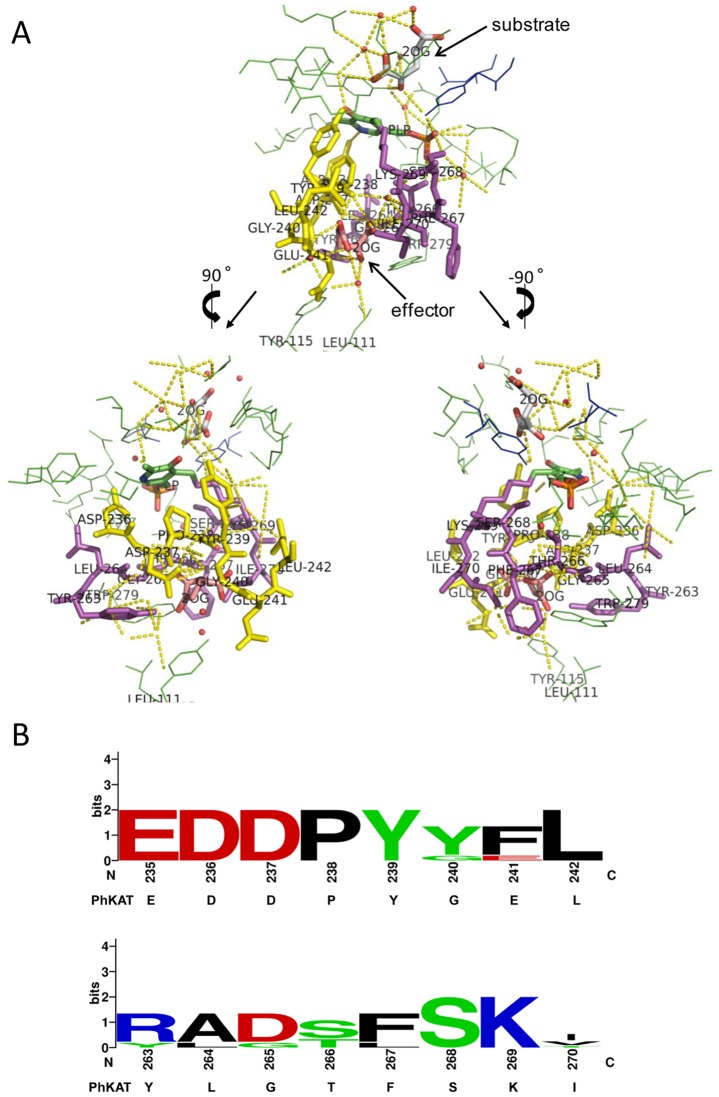
Close-up view of an allosteric effector-binding site and its sequence logo. (A) Line-and-stick representations of the binding sites of PLP and 2OG molecules as a cofactor, substrate, and allosteric effector. Black arrows show 2OG in its positions as a substrate (upper) and allosteric effector (lower). Lower sites are rotated 90° and −90° relative to the upper site. The figure was generated using PyMOL. (B) Sequence logo of the allosteric effector-bound site. Upper, yellow stick representation region of (A) + Glu-235; lower, magenta stick representation region of (A). “K-269” is a ligand bound with a C4A atom of PLP. Amino acids are colored according to their chemical properties: polar amino acids (G, S, T, and Y) are green, basic (K and R) blue, acidic (D and E) red, and hydrophobic (V, A, L, I, P, and F) amino acids are black. The protein sequence alignment used mammalian KAT IIs and PhKAT. GenBank database (GB) accession numbers for *P. horikoshii*, humans (*Homo sapiens*), rats (*Rattus norvegicus*), mice (*Mus musculus*), pigs (*Sus scrofa*), and monkeys (*Macaca mulatta*) are NP_142204, NP_057312, NP_058889, NP_035964, XP_001924647, and XP_002804303, respectively. Multiple alignments were performed using ClustalW2. The sequence logo was generated using WebLogo [22].

To verify that PhKAT and 2OG are an allosteric enzyme and allosteric effector, respectively, we cocrystallized PhKAT, PLP, and 2OG in a 1∶1:2 ratio. PhKAT has 4 2OG-binding sites that have different binding characters. In the present study, we determined previously unknown allosteric sites of PhKAT (Fig. 8). The allosteric sites of PhKAT are located on the back of the active sites, and PLP is interleaved between the active and allosteric sites (Fig. 8A). The allosteric sites are comprised of the Asp-236–Leu-242, Tyr-263–Ile-270, Leu-111, Tyr-115, and Trp-279 residues (Fig. 8A). The allosteric sites are communalized with a PLP-binding site (Fig. 8A and B). Glu-235–Tyr-239, Leu-242, Ser-268, and Lys-269 are essentially conserved between PhKAT and mammalian KAT IIs (e.g., humans, rats, mice, pigs, and monkeys) (Fig. 8B). The Glu-235–Leu-242 region was more conserved than the Tyr-263–Ile-270 region. The results indicate that allosteric sites may be conserved in mammalian KAT II families.

## Discussion

The results of this study show that the mechanisms of KYNA synthesis from KYN is conserved in the hyperthermophilic archaeon, *P. horikoshii*, and that KYN transamination results in the production of KYNA (Figs. 3 and 4). This suggests that although the amino-acid sequence of PhKAT is quite different from that of HuKAT II, the metabolic conversion of KYN to KYNA is conserved. Consistent with this, the crystal structures of the PhKAT–PLP complex and PhKAT–PLP–2OG triple complex, which forms a necessary bio-functional complex that catalyzes the formation of KYNA from KYN, are similar to those of HuKAT II [13], [14] (Fig. 2). The proposed mechanism of the KYNA synthesis reaction from KYN in conjunction with 2OG and/or OXA is depicted in Figure S8.

However, not all aspects of KYNA synthesis are conserved; one factor that is highly variable between different groups of organisms is the source of transaminating acceptor equivalents. The specificity of transaminated acceptors among various organisms is not fairly high for KAT enzymes. Therefore, to determine an authentic substrate as acceptors, and/or allosteric effector for PhKAT, we probed the interaction between 2OG and a KAT protein using ITC. The results show that 2OG binds PhKAT with high specificity and affinity (Fig. 5). This scheme strongly indicates that the KYNA biosynthesis pathway may be linked with the 2OG biosynthetic pathway. In fact, *P. horikoshii* possesses the pathway.

Another important feature of PhKAT is regulated by its dependence on changes in the concentration of 2OG. To assess whether the proteins of *P. horikoshii*, humans, or other mammals are biochemically conserved, we determined the substrate requirements for the *P. horikoshii* enzyme. The results show that PhKAT activity for converting KYN to KYNA is supported by 2OG and OXA molecules (Fig. 4). Moreover, the results show that PhKAT activity is regulated by allosteric control by 2OG (Fig. 4A and B). Our studies state that *in vivo*, KAT activities are accelerated when 2OG and/or OXA concentrations are low and vice versa (Fig. 4A, B, E and F). The high thermostabilities of murine KAT families were recently reported [16]. Therefore, this implicitly indicates that the regulation systems of PhKAT might be evolutionarily conserved between hyperthermophilic archaea and mammals including humans.Some factors that affect enzymatic activity are electrostatic and hydrophobic substrate interactions, overall dipole moments of enzymes, certain aromatic groups in the KYNA biosynthesis pathway, and the relative orientation and distance between prosthetic groups in the complex. The interactions between PLP cofactor, 2OG, OXA and KYN substrates, and KAT mainly occur as a result of electrostatic interactions. The present study elucidates the binding mechanisms of substrates, cofactors, and PhKAT using spectrophotometry and ITC. The substrate, cofactor, and PhKAT complexes were formed in the reaction, suggesting the existence of cooperation between KAT and KYN and/or 2OG. The regulation of cooperative and binding mechanisms was discovered through the conversion activity of KYNA and the formation of substrate, cofactor, and PhKAT complexes. With coexisting substrates, the binding affinities of 2OG for PLP–KAT complexes change to decrease or increase. It should be possible to determine the binding affinity of the KYNA conversion reaction in real time.

We revealed the characteristic features of KAT from a hyperthermophilic archaeon, *P. horikoshii*, using enzyme analysis. In this study, we identified PhKAT as an allosteric enzyme regulated by 2OG in cooperation with KYN. The results indicate that the conversion reaction from KYN to KYNA by PhKAT is regulated depending on the concentration of 2OG as a transamination acceptor. In addition, KYN may be an activator for PhKAT in order to unlock an inhibiting action by 2OG. 2OG and OXA may function as efficient transamination acceptors for KAT in *P. horikoshii* OT3 because it exhibits high affinities for PhKAT as detected by kinetics and/or ITC. In addition, PhKAT exhibits a high affinity for PLP. These findings indicate that high affinities may be required for KAT protein functioning in extreme environments hotter than 100°C.

To obtain more knowledge about the function of KAT and evidence to support our findings that the crystal structure of PhKAT in complex with an allosteric effector (2OG) is very important, we decided to investigate the complex formation between PhKAT and 2OG. For this purpose, we cocrystallized both intact PhKAT and 2OG molecules, which are required for enzymatic regulation *in vivo*, and acquired a bio-functional complex. The results are in complete agreement with our investigation, demonstrating that 2OG as a substrate is critical as the rate-limiting factor in the KYNA biosynthetic pathway.

Finally, we suggest that the KAT protein from *P. horikoshii* OT3 may be evolutionarily conserved and related to human KATs localized in the brain. The enzymatic molecular mechanisms for the conversion reaction from KYN to KYNA by PhKAT remain unclear. Therefore, we solved the crystal structures of the PhKAT complexed with KYN or PLP to understand the differences in the enzymatic reaction mechanisms between human KATs and PhKAT in greater detail [13], [14], [17]. The present study clearly demonstrates that 2OG is an allosteric inhibitor that binds the allosteric sites of PhKAT. We propose that 2OG as a substrate regulates the KYNA biosynthesis of PhKAT *in vivo* and that PhKAT shares a function of 2OG and has 4 pockets with different binding affinities for 2OG. Thus, the allosteric sites of KAT may be a novel drug target for the regulation of KYNA production.

## Methods

### Reagents

Pyridoxal phosphate (Wako), l-kynurenine (SIGMA), kynurenic acid (SIGMA), 2-oxoglutaric acid (2OG; Wako), oxaloacetic acid (OXA; Wako), 2-ketobutyric acid (α-KBA; ALDRICH), and α-keto-γ-(methylthio) butyric acid (α-KMB; SIGMA) preparations were used as a cofactor, and substrates and/or standards; agar and organic nutrients for Luria–Bertani (LB) medium (Difco) were purchased. PLP, KYN, and KYNA were treated with alkali to dissolve them in water.

### Construction of the pET28a-PhKAT Expression Vector

The pET28a-PhKAT expression vector for the expression of His6-tagged PhKAT was constructed by ligating the *Nde*I–*Eco*RI fragment from pET1300Ph [15] into the *Nde*I–*Eco*RI sites of pET28a (Novagen).

### Expression and Purification of Recombinant PhKAT

A single fresh colony of *E. coli* BL21-CodonPlus (DE3) transformed with the pET28a-PhKAT was cultured in 50 mL LB medium containing kanamycin (30 µg/mL) overnight at 37°C. Twenty milliliters of this culture was inoculated into 2 L LB medium and cultivation was continued until the optical density at 578 nm (OD_578_) reached 0.6. Protein expression was induced by the addition of 0.3 mM isopropylthio-β-galactoside (IPTG) and incubation for 21 h at 17°C; the bacteria were subsequently harvested by centrifugation. The bacterial pellets from 2 L culture were resuspended in 30 mL lysis buffer (50 mM sodium phosphate [pH 7.0], 1 mM dithiothreitol [DTT], and 300 mM NaCl), and the cells were lysed by 30-s sonication on ice. Cell debris was removed by centrifugation at 43 000 × *g* for 30 min. The resultant supernatant was loaded directly onto a His TALON Cartridge (1 mL; Clontech Laboratories) equilibrated with 10 column volumes of lysis buffer at 4°C using a syringe. Unbound protein was eliminated by washing the column with 10 column volumes of lysis and wash buffer (50 mM sodium phosphate [pH 7.0], 300 mM NaCl, and 5 mM imidazole). PhKAT was eluted with 5 column volumes of elution buffer (50 mM sodium phosphate [pH 7.0], 300 mM NaCl, and 150 mM imidazole). Fractions containing PhKAT were pooled and concentrated to 1 mL using an Amicon Ultra with a 30-kDa cutoff (Millipore). This fraction was rebuffered in 50 mM HEPES–NaOH buffer (pH 7.5) containing 100 mM NaCl for the enzymatic assay and ITC or 5 mM HEPES–NaOH buffer (pH 7.5) using prepacked Sephadex G-25 gel filtration columns NAP-10 (GE Healthcare UK Ltd.) for crystallization. The protein concentration of monomeric PhKAT was determined spectroscopically with an extinction coefficient of 55,810 M^−1^⋅cm^−1^ at 280 nm.

### Spectroscopic Analysis of PLP Binding

PhKAT was added to a 20 µM (final concentration) PLP solution in a final volume of 500 µL of 50 mM HEPES–NaOH buffer (pH 7.5) containing 100 mM NaCl. Titration of PhKAT with PLP was monitored by absorption spectroscopy. Aliquots of PLP (1, 2, 4, 8, 16, 32, and 64 µM) were added to the cuvette containing PhKAT at 25°C. Spectra were recorded after the addition of PLP, and the PLP-binding constant (*K*_d_) was calculated from the difference in absorbance at 360 nm, and the PLP-binding curve for PhKAT was obtained by the 2-site binding model with variable slopes using prism5.

### Enzyme Activity Assay and PhKAT Kinetics

PLP-dependent KAT activity was assayed in 500 µL of 50 mM HEPES–NaOH buffer (pH 7.5) containing 100 mM NaCl, 10 µM PhKAT, 15 µM PLP, 20 µM KYN, and 64 µM 2OG. Assays were performed at room temperature (25°C), and the reaction was initiated by the addition of 2OG. KYNA production was monitored spectrophotometrically using a U-2810 UV–Vis spectrophotometer (Hitachi).

The transaminase abilities of KAT were assayed by measuring the rate of the KYNA production. In a total volume of 500 µL, the reaction mixture contained 10 µM PhKAT (dimer: 5 µM); 12 µM PLP; 0.08, 0.1, 0.4, 0.8, 1, 1.2, 2, 4, 6, 8, 12, 16, 20 and 24 µM 2OG; 0.02, 0.08, 0.1, 0.4, 0.8, 1, 1.2, 2, 4, 6, 8, 12, 16, 20 and 24 µM OXA; 50, or 100 µM KYN; 50 mM HEPES–NaOH buffer (pH 7.5); 100 mM NaCl; 0.4, 0.8, 1, 4, 8, 12 and 16 µM α-KMB, and/or 0.4, 0.8, 1, 4, 8, 12, 16 and 20 µM α-KBA. KYNA production was monitored by the increase in A332 at 25°C. The quantities of KYNA were determined spectroscopically with an extinction coefficient of 9.8 mM^−1^⋅cm^−1^ at 332 nm.

### Isothermal Titration Calorimetry for Cofactor and Substrate Binding

The binding affinities of PhKAT with cofactor (PLP) and substrate (2OG) were determined by high-sensitivity microcalorimetry using a VP-ITC device (GE Healthcare UK Ltd.) at 35°C. To avoid air bubbles, we degassed solutions under vacuum before use. Fresh PhKAT, and/or PLP and KYN were filled in the reaction cell at concentrations of 20, 30, or 40 µM (dimer: 10, 15, and 20 µM) in 50 mM HEPES–NaOH buffer (pH 7.5) containing 100 mM NaCl and titrated in 25 10-µL steps against stock solutions of PLP (0.3 mM), 2OG (0.2 or 0.4 mM) at 5-min intervals. Table S4 summarizes the ITC control parameters. Power peaks were integrated, and the resultant reaction temperatures were plotted against the molar cofactor and/or substrate/protein ratios and fitted using the “sequential binding sites” model according to the manufacturer’s instructions with origin version 5.0 (MicroCal Software), thus yielding the dissociation constant *K*_d_.

### Cocrystallization

We searched extensively for the crystallization conditions of the PLP and/or 2OG complexes with PhKAT using the sitting-drop vapor diffusion method with Crystal Screen (CS) I and II (Hampton Research) and Wizard (Wiz) I and II (Emerald Biosystems). In brief, PhKAT was concentrated to 15 mg/mL (306.6 µM) and mixed with PLP and/or 2OG at 1∶1, and 1∶1:1 or 1∶1:2 ratios, respectively. Crystals of KAT complexes with cofactors and/or substrates and/or allosteric effectors were grown using the sitting-drop vapor diffusion method in CS I No. 14 (0.2 M calcium chloride dehydrate, 0.1 M HEPES sodium [pH 7.5], 28% v/v polyethylene glycol [PEG] 400) for the PLP–2OG–PhKAT complex, CS I No. 23 (0.2 M magnesium chloride hexahydrate, 0.1 M HEPES sodium [pH 7.5], 30% v/v PEG 400) for the PLP–PhKAT complex, and Wiz I No. 12 (0.1 M imidazole [pH 8.0], 0.2 M calcium acetate, 20% w/v PEG 1000) for the allosteric effector complex at 4°C.

### Crystallographic Data Collection and Processing

The crystals were soaked in crystallization solution containing an additional 18% ethylene glycol (vol/vol) as a cryoprotectant for the allosteric effector complex (PEG400 becomes a cryoprotectant). Radiographic data for the allosteric effector complex were collected at wavelengths of 0.9 or 1 Å at beam line BL38B1 at the SPring-8 synchrotron in Hyogo, Japan. Diffraction images were collected on a CCD-based detector system (ADSC Quantum 210) at liquid nitrogen temperature (100 K). Diffraction data were indexed, integrated, and scaled using the HKL2000 program suite. Crystal data and crystallographic statistics are shown in Table S1.

### Structural Determination and Refinement

The structures were solved via the molecular replacement method, using the structure of apo-PhKAT (PDB cord: 1×0M) or PLP complex (PDB cord: 3AOV) as a search model with the MOLREP program [18] in the CCP4i program suite [19]. First, the structures of PhKAT complexes were refined to rigid bodies and refined again with isotropic temperature factors by using the REFMAC5 program [20]. The atomic models were manually revised using Coot [21]. Upon careful inspection of the |F_o_|–|F_c_| and 2|F_o_|–|F_c_| maps, we added PLP, water molecules, and/or 2OGs into the model. Subsequent refinement was done at resolutions of up to 1.72, 1.56, and 1.69 Å. The stereochemical geometry of the model was checked with the PROCHECK program.

### Accession Codes

The atomic coordinates and structure factors for PhKAT in complex with PLP and 2OG as substrates and/or allosteric effectors have been deposited in the Protein Data Bank with the following accession codes: PLP, 3AOV; PLP and 2OGs as substrate, 3AOW; PLP and 2OGs as substrate and allosteric effector, 3ATH.

## Supporting Information

Figure S1 **Scheme of the KAT-catalyzed reaction showing the conversion of KYNA from KYN.** KYNA is synthesized from KYN via a 4AD intermediate.
(TIF)

Figure S2 **Spectrophotometric assay of** **the cofactor binding to** **PhKAT.** PhKAT binds with the PLP cofactor as measured by absorbance spectroscopy. (A), PLP only, (B), 10 µM PhKAT, (C), 20 µM PhKAT. PLP performed at concentrations of 1, 2, 4, 8, 16, 32 and 64 µM.
(TIF)

Figure S3 **Spectrophotometric assay of the time course of the PhKAT-catalyzed activity of KYN.** The spectrum changes were monitored after the addition of 2OG, and PhKAT. 7 µM PLP; 20 µM KYN; 1 mM 2OG; 2 µM PhKAT. The enzymatic activity of PhKAT for KYNA productions cannot measure at this condition.
(TIF)

Figure S4 **Comparison between the sequences of PhKAT and HuKAT II by pairwise alignment.** The alignment was created using BioEdit and ESPript. Ph, *Pyrococcus horikoshii*; Hu, human. Black arrows and cylinders indicate the β-sheets and α-helices, respectively. Identical residues are in red boxes. A blue arrowhead indicates a PLP ligand lysine. The horizontal line indicates a possible mitochondrial-targeting peptide (MTP) of HuKAT II predicted by TargetP. PhKAT shares 27% identity with HuKAT II. The GenBank database (GB) accession numbers for PhKAT and HuKAT II are NP_142204 and NP_057312, respectively.
(TIF)

Figure S5 **Allosteric regulations for PhKAT.** (A) The sigmoid dose-response shows the allosteric inhibition by a second allosteric effector. The region of 8∼24 µM 2OG (data: means, n = 5) in Figure 4A is performed a curve fitting using a dose-response model with variable slopes for the inhibition and an altered equation of Cheng and Prusoff (Equ. S2). The absolute inhibition constant (*K*_i_) of 2OG for PhKAT was 20.11 µM (Table S2.). (B) Close-up views of ∼2 µM 2OG regions in Figure 4A and B. A black arrow and arrow heads indicate the conformation change from R state to T state of PhKAT and allosteries for KYNA productions respectively. Black and white arrow heads indicate the inhibition by 2OG and activation by KYN, respectively. A max velocity at 1.2 µM 2OG of right hand increased 60% more than left hand.
(TIF)

Figure S6 **ITC controls for the interaction between the cofactor, substrates and KAT.** The ITC profiles include experimental conditions. (A) buffer and PLP; (B) PLP and 2OG; (C) PLP-KYN and 2OG.
(TIF)

Figure S7 **Superimposed representation of PLP and/or 2OG as substrate- and allosteric effector-bound complexes structures of PhKAT.** Close-up view and Cα-trace ribbon-and-line representation of the α2-helix of PLP complex (PDB code: 3AO V) and 2OG effector complex structures of PhKAT after optimal superimposition. The PLP-bound PhKAT complex is colored gray. Black-labeled numbers indicate the distance (Å) between the Cα traces of PLP complex and allosteric effector complex structures. The figure was generated using PyMOL.
(TIF)

Figure S8 **The proposed mechanism for KYNA synthesis from KYN mediated by PhKAT.** KYNA is synthesized from KYN via sequential reactions of KAT. PhKAT transaminates KYN to 2OG and/or OXA via PMP. OXA and/or 2OG function as an amino-group acceptors and a naturally allosteric inhibitor that regulates KAT activity, respectively. GLU, glutamic acid; ASP, asparatic acid.
(TIF)

Table S1 **Data collection and refinement statistics for PhKAT in complex with PLP and/or 2OG.**
(TIF)

Table S2 **Comparison of the kinetic parameters of substrates for the transaminase reaction from KYN to the keto-acid group.** The transamination abilities of KYN to acceptors (2OG and α-keto-analogs) were assayed by measuring the rate of KYNA production.
(TIF)

Table S3 **ITC parameters of cofactor and substrate binding to PhKAT.** All measurements were performed in 50 mM HEPES–NaOH buffer (pH 7.5) with 100 mM NaCl.
(TIF)

Table S4 **ITC control parameters.**
(TIF)

Equation S1 **Two sites binding with hill slopes (altered two sites binding model).**
(TIF)

Equation S2 **Sigmoidal dose-response and absolute inhibition constant (** ***K*** **_i_).** The IC50 value was converted to an absolute inhibition constant *K*_i_ using the altered Cheng-Prusoff equation.
(TIF)

Equation S3 **Allosteric sigmoidal.**
(TIF)
