# Supplementary figures and images for: Characteristic Features of Kynurenine Aminotransferase Allosterically Regulated by (Alpha)-Ketoglutarate in Cooperation with Kynurenine
Source: PLoS One. 2012 Jul 6;7(7):e40307. doi: 10.1371/journal.pone.0040307 (PMC3391261; doi:10.1371/journal.pone.0040307)

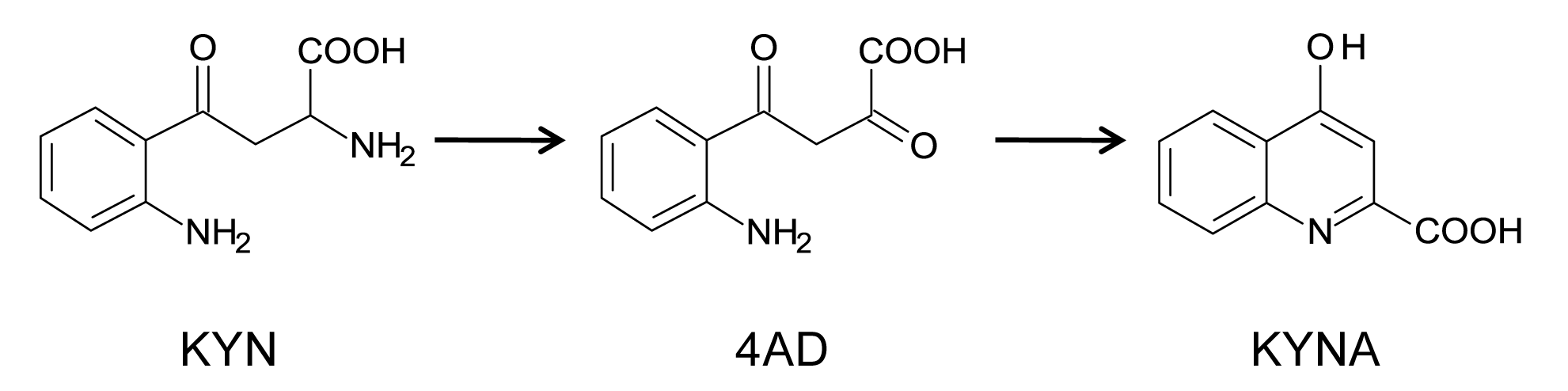

Supplement: Figure S1 — Scheme of the KAT-catalyzed reaction showing the conversion of KYNA from KYN. KYNA is synthesized from KYN via a 4AD intermediate. (TIF) [file pone.0040307.s001.tif]

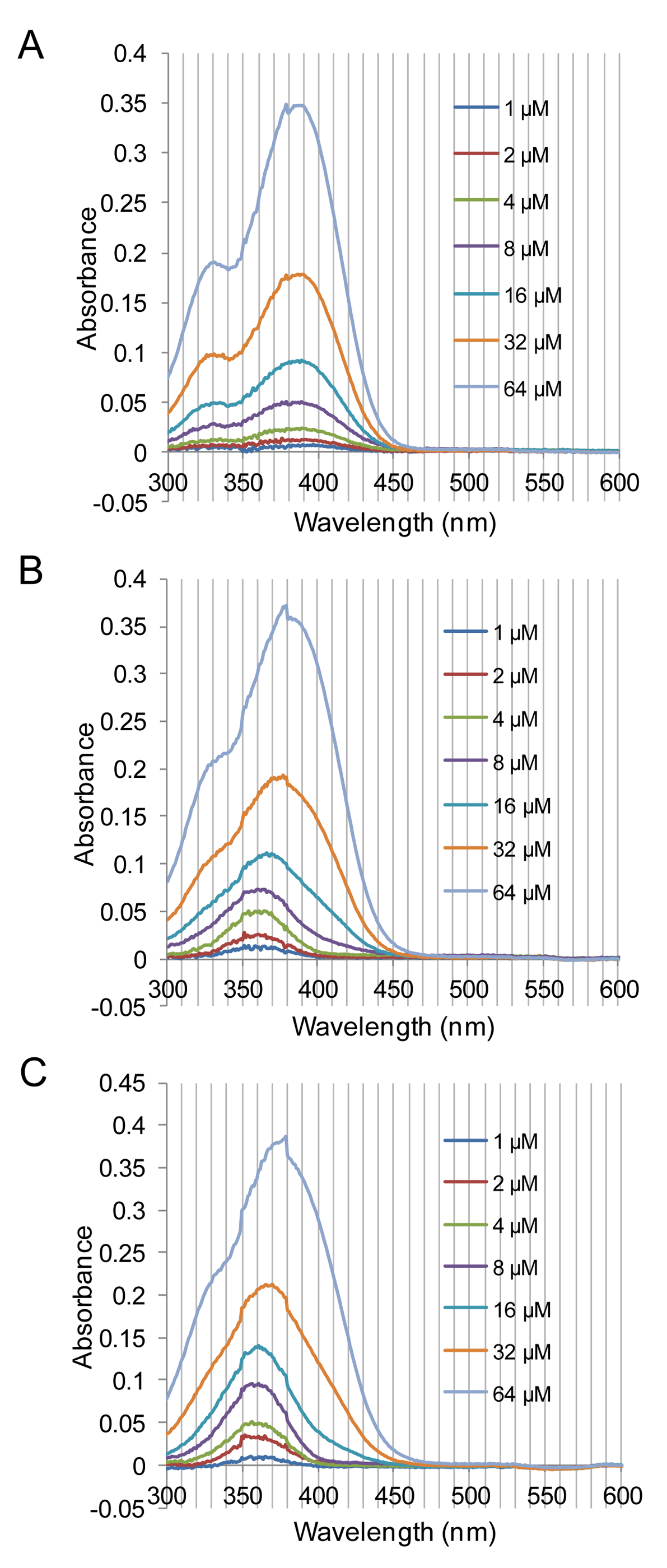

Supplement: Figure S2 — Spectrophotometric assay of the cofactor binding to PhKAT. PhKAT binds with the PLP cofactor as measured by absorbance spectroscopy. (A), PLP only, (B), 10 µM PhKAT, (C), 20 µM PhKAT. PLP performed at concentrations of 1, 2, 4, 8, 16, 32 and 64 µM. (TIF) [file pone.0040307.s002.tif]

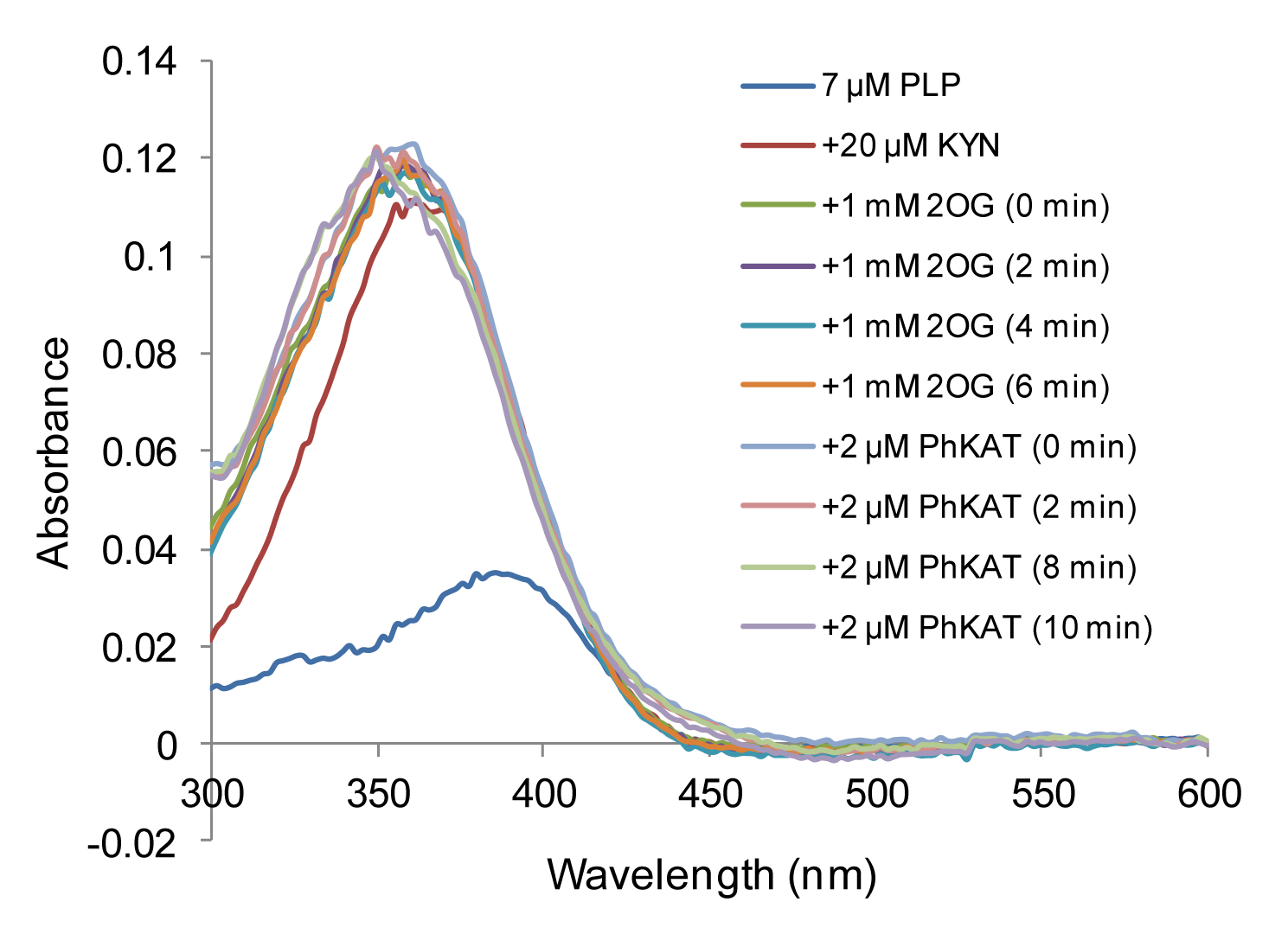

Supplement: Figure S3 — Spectrophotometric assay of the time course of the PhKAT-catalyzed activity of KYN. The spectrum changes were monitored after the addition of 2OG, and PhKAT. 7 µM PLP; 20 µM KYN; 1 mM 2OG; 2 µM PhKAT. The enzymatic activity of PhKAT for KYNA productions cannot measure at this condition. (TIF) [file pone.0040307.s003.tif]

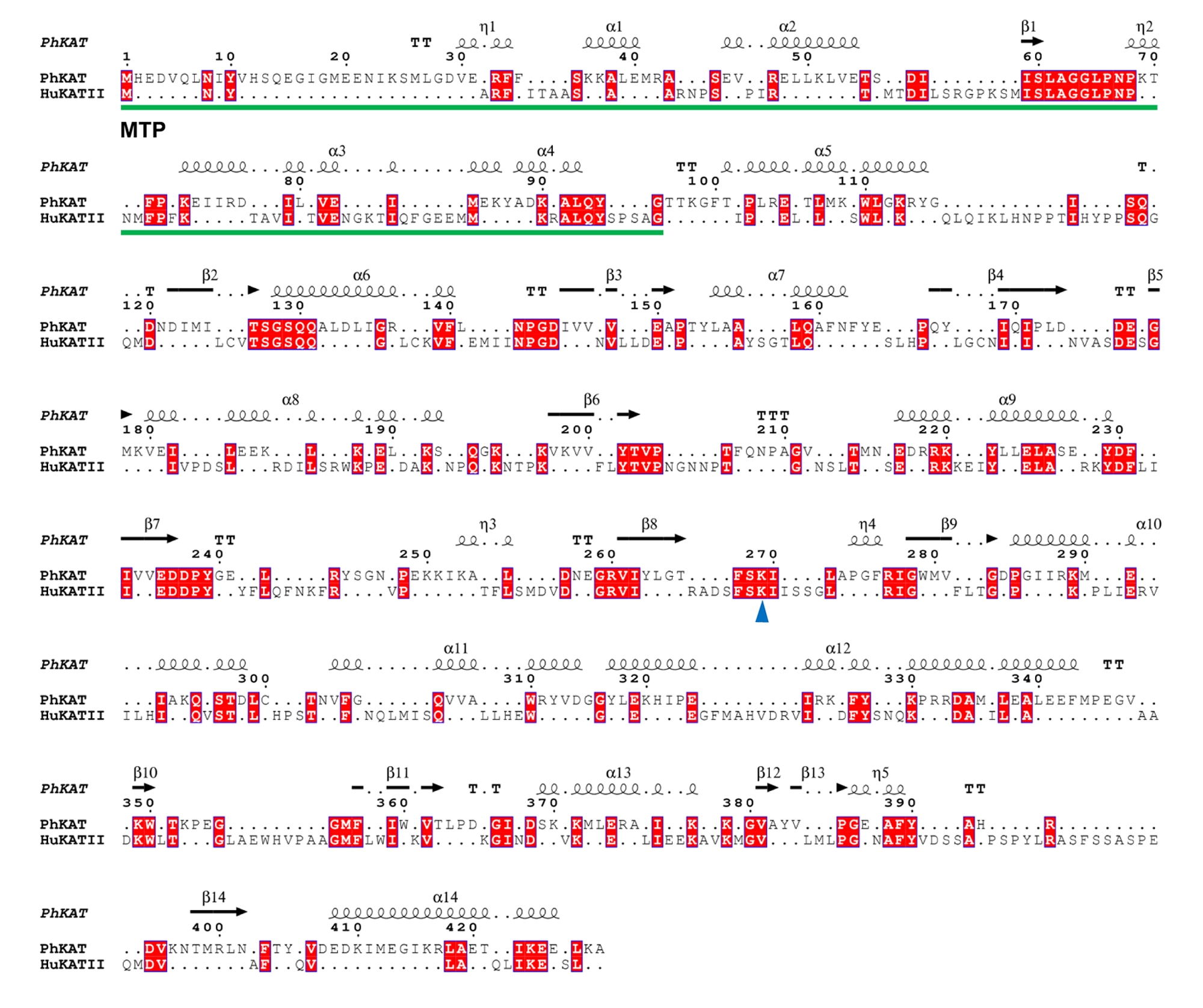

Supplement: Figure S4 — Comparison between the sequences of PhKAT and HuKAT II by pairwise alignment. The alignment was created using BioEdit and ESPript. Ph, Pyrococcus horikoshii; Hu, human. Black arrows and cylinders indicate the β-sheets and α-helices, respectively. Identical residues are in red boxes. A blue arrowhead indicates a PLP ligand lysine. The horizontal line indicates a possible mitochondrial-targeting peptide (MTP) of HuKAT II predicted by TargetP. PhKAT shares 27% identity with HuKAT II. The GenBank database (GB) accession numbers for PhKAT and HuKAT II are NP_142204 and NP_057312, respectively. (TIF) [file pone.0040307.s004.tif]

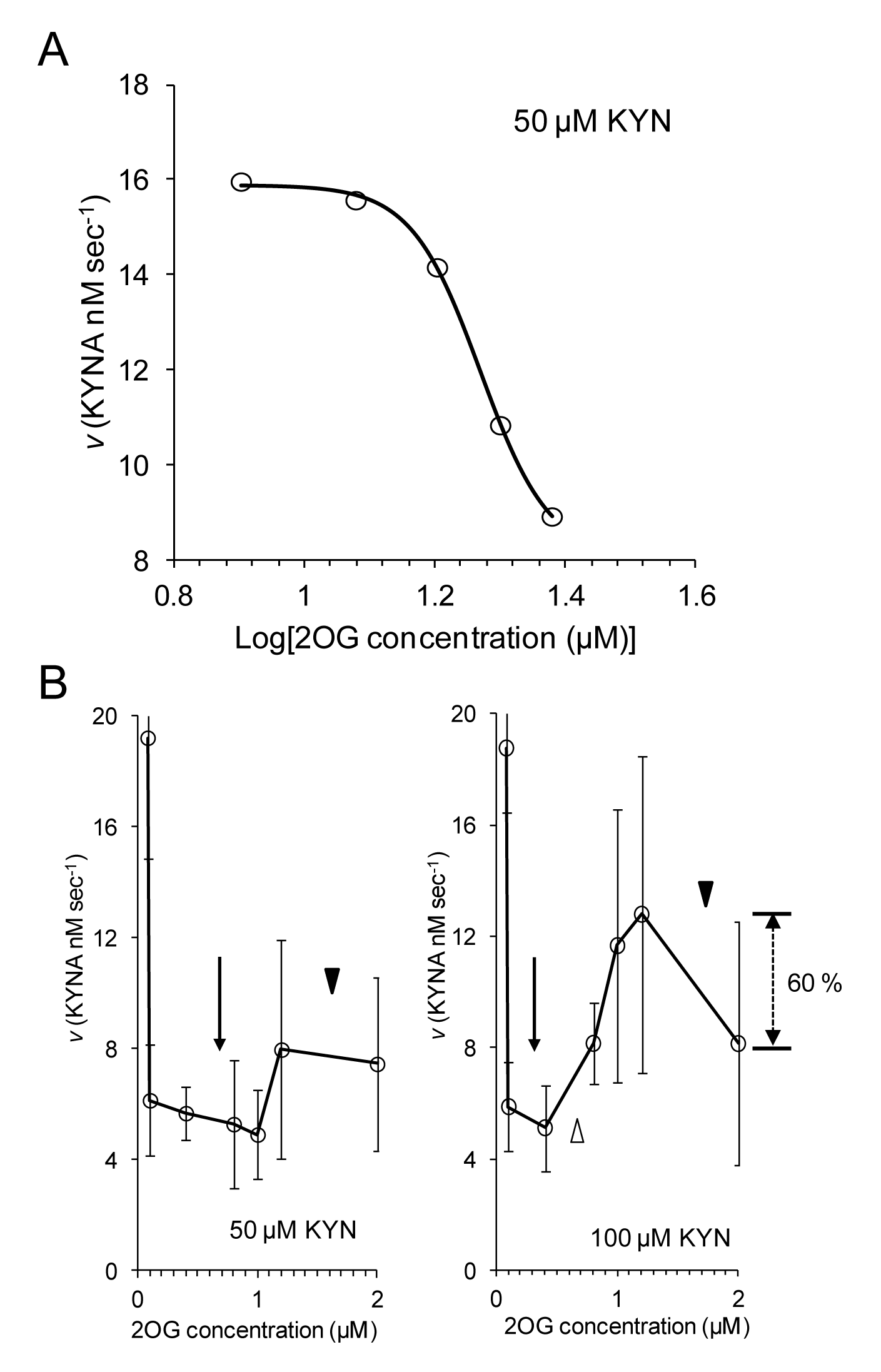

Supplement: Figure S5 — Allosteric regulations for PhKAT. (A) The sigmoid dose-response shows the allosteric inhibition by a second allosteric effector. The region of 8∼24 µM 2OG (data: means, n = 5) in Figure 4A is performed a curve fitting using a dose-response model with variable slopes for the inhibition and an altered equation of Cheng and Prusoff (Equ. S2). The absolute inhibition constant (K i) of 2OG for PhKAT was 20.11 µM (Table S2.). (B) Close-up views of ∼2 µM 2OG regions in Figure 4A and B. A black arrow and arrow heads indicate the conformation change from R state to T state of PhKAT and allosteries for KYNA productions respectively. Black and white arrow heads indicate the inhibition by 2OG and activation by KYN, respectively. A max velocity at 1.2 µM 2OG of right hand increased 60% more than left hand. (TIF) [file pone.0040307.s005.tif]

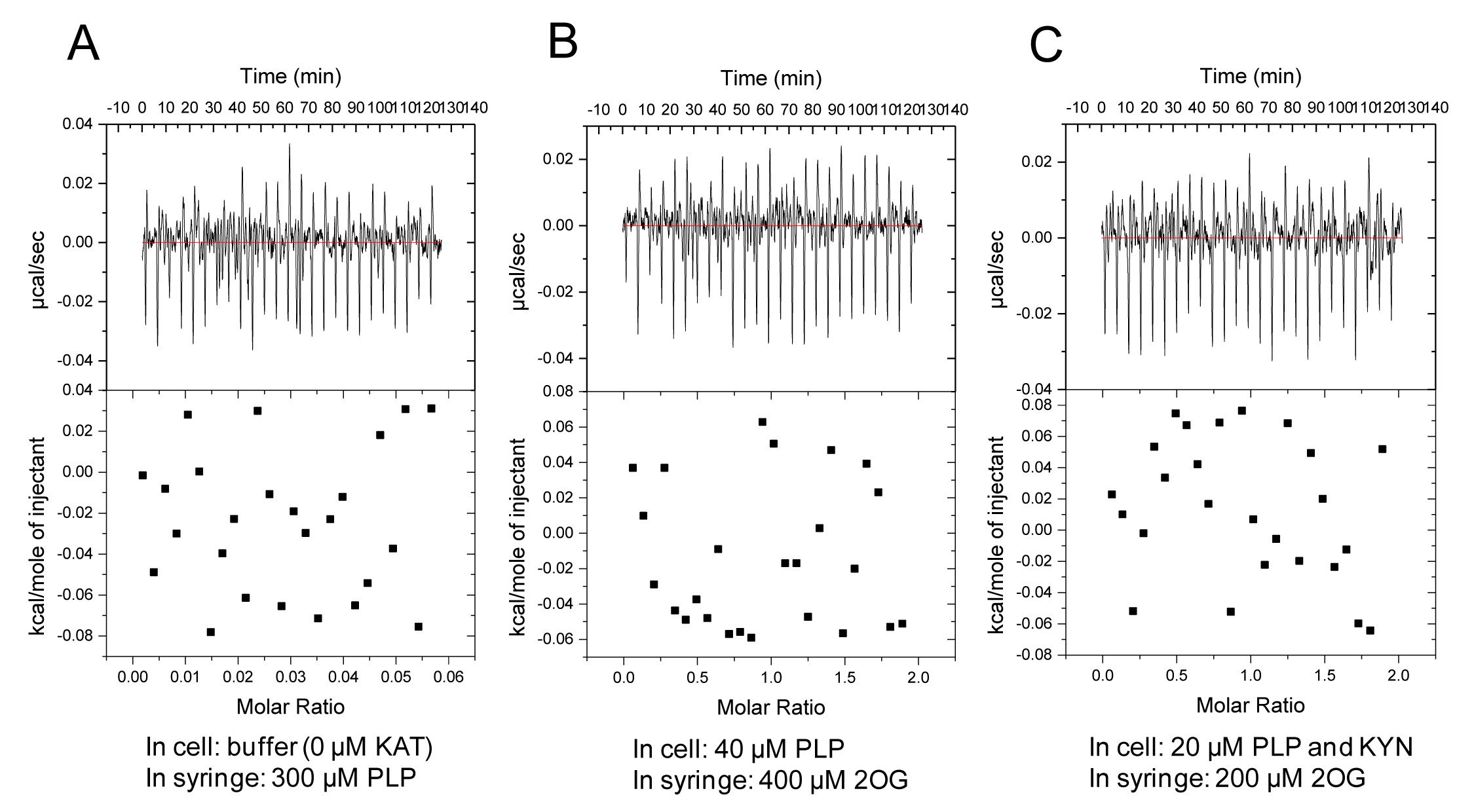

Supplement: Figure S6 — ITC controls for the interaction between the cofactor, substrates and KAT. The ITC profiles include experimental conditions. (A) buffer and PLP; (B) PLP and 2OG; (C) PLP-KYN and 2OG. (TIF) [file pone.0040307.s006.tif]

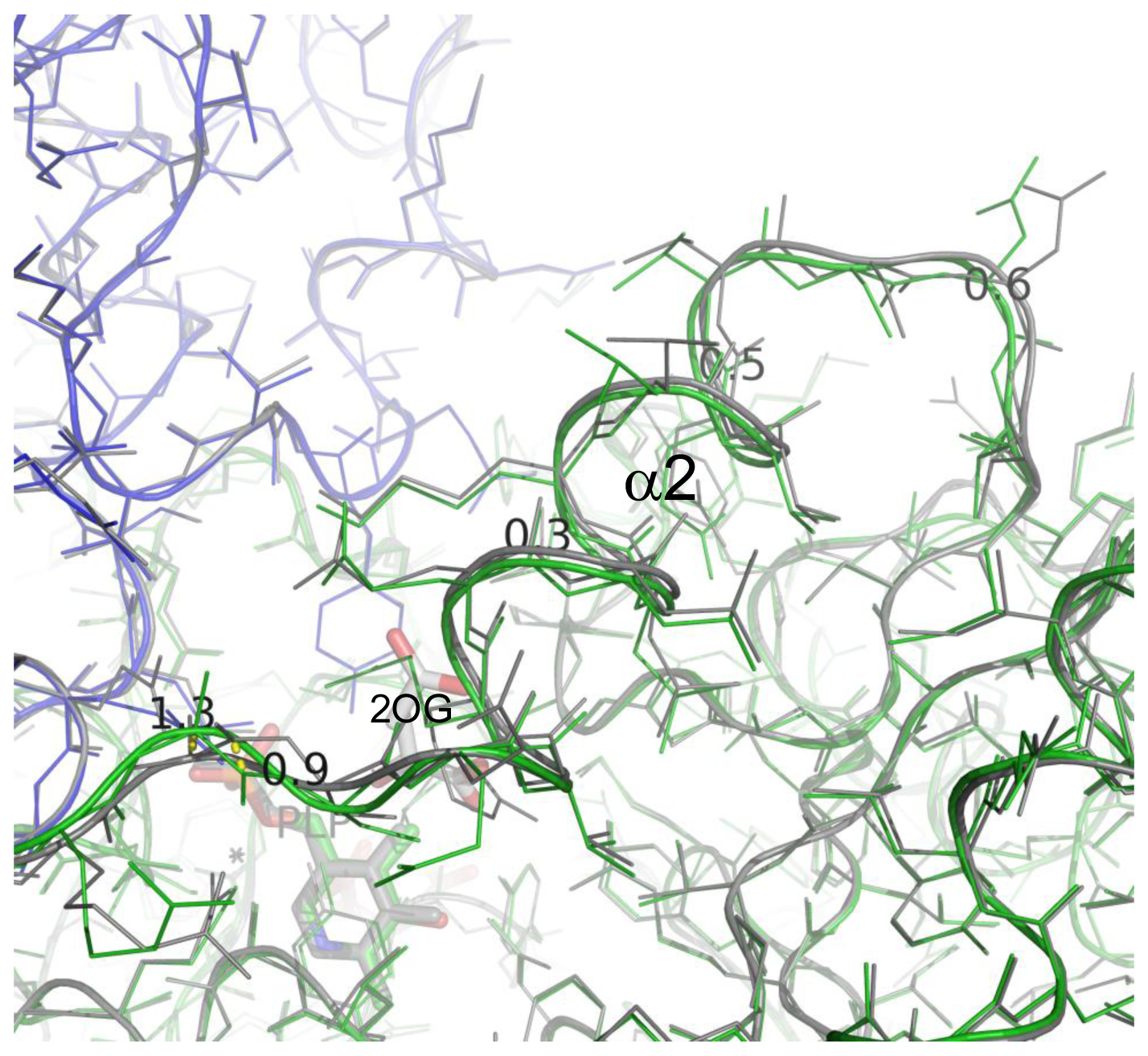

Supplement: Figure S7 — Superimposed representation of PLP and/or 2OG as substrate- and allosteric effector-bound complexes structures of PhKAT. Close-up view and Cα-trace ribbon-and-line representation of the α2-helix of PLP complex (PDB code: 3AO V) and 2OG effector complex structures of PhKAT after optimal superimposition. The PLP-bound PhKAT complex is colored gray. Black-labeled numbers indicate the distance (Å) between the Cα traces of PLP complex and allosteric effector complex structures. The figure was generated using PyMOL. (TIF) [file pone.0040307.s007.tif]

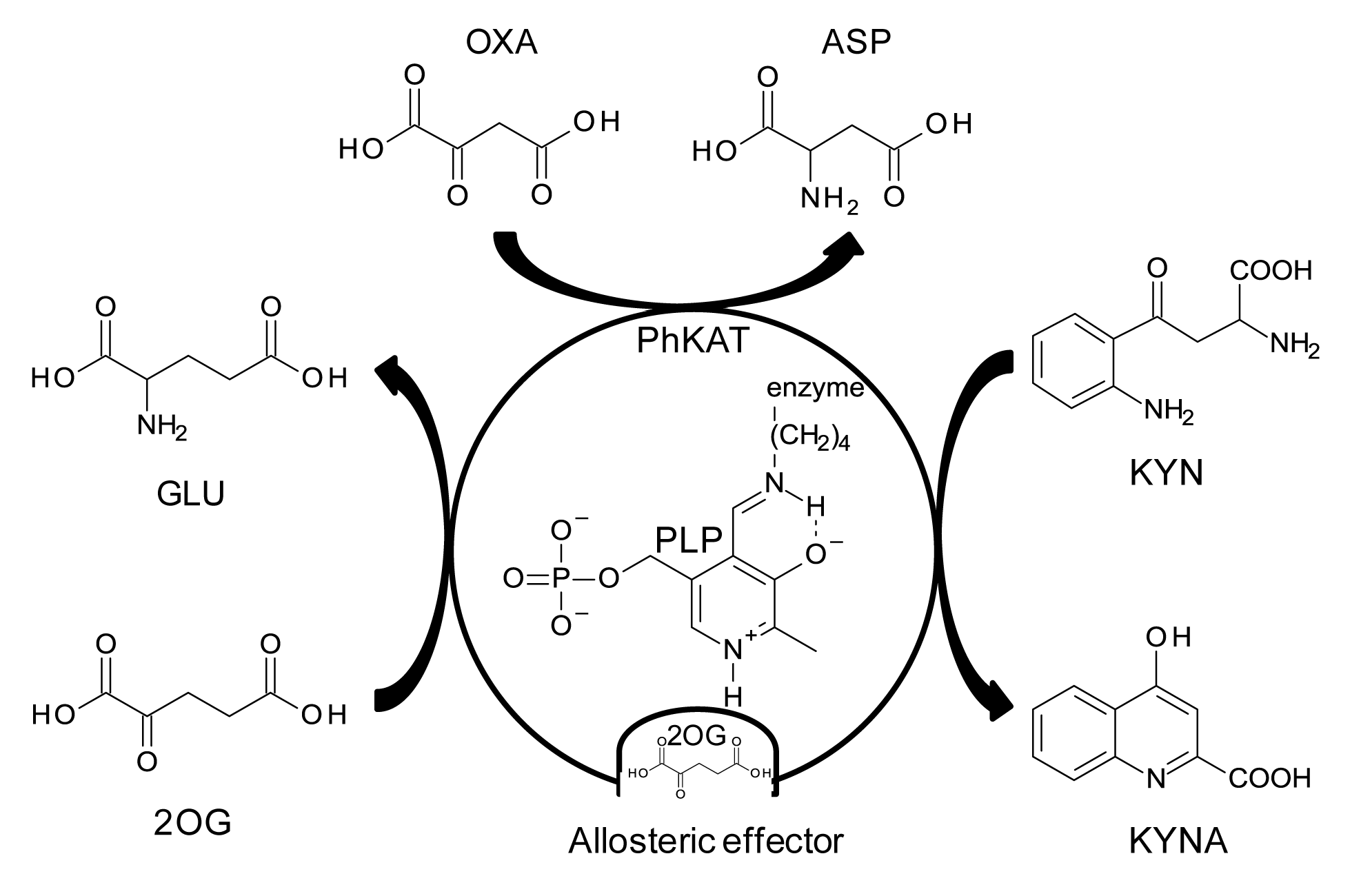

Supplement: Figure S8 — The proposed mechanism for KYNA synthesis from KYN mediated by PhKAT. KYNA is synthesized from KYN via sequential reactions of KAT. PhKAT transaminates KYN to 2OG and/or OXA via PMP. OXA and/or 2OG function as an amino-group acceptors and a naturally allosteric inhibitor that regulates KAT activity, respectively. GLU, glutamic acid; ASP, asparatic acid. (TIF) [file pone.0040307.s008.tif]

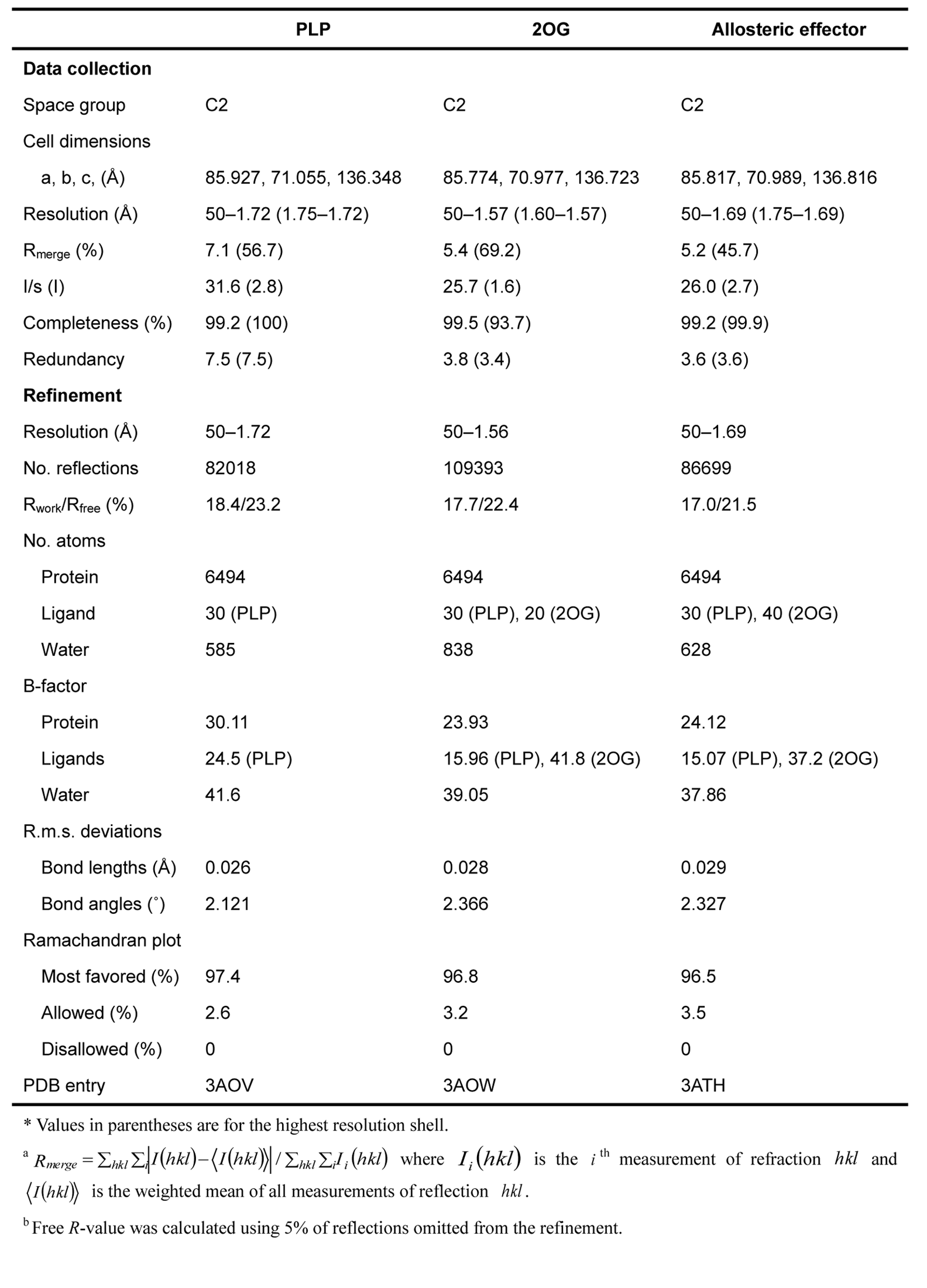

Supplement: Table S1 — Data collection and refinement statistics for PhKAT in complex with PLP and/or 2OG. (TIF) [file pone.0040307.s009.tif]

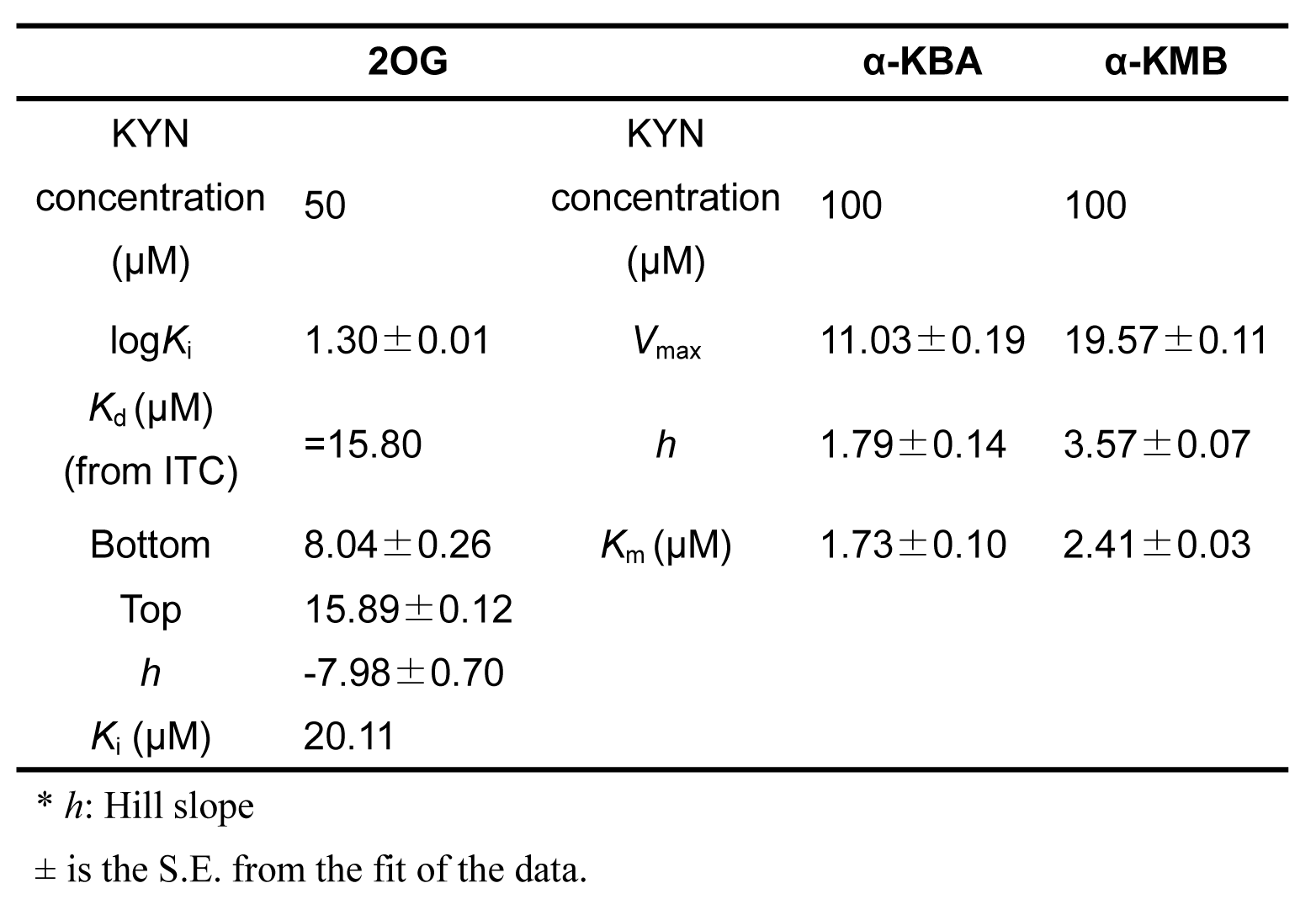

Supplement: Table S2 — Comparison of the kinetic parameters of substrates for the transaminase reaction from KYN to the keto-acid group. The transamination abilities of KYN to acceptors (2OG and α-keto-analogs) were assayed by measuring the rate of KYNA production. (TIF) [file pone.0040307.s010.tif]

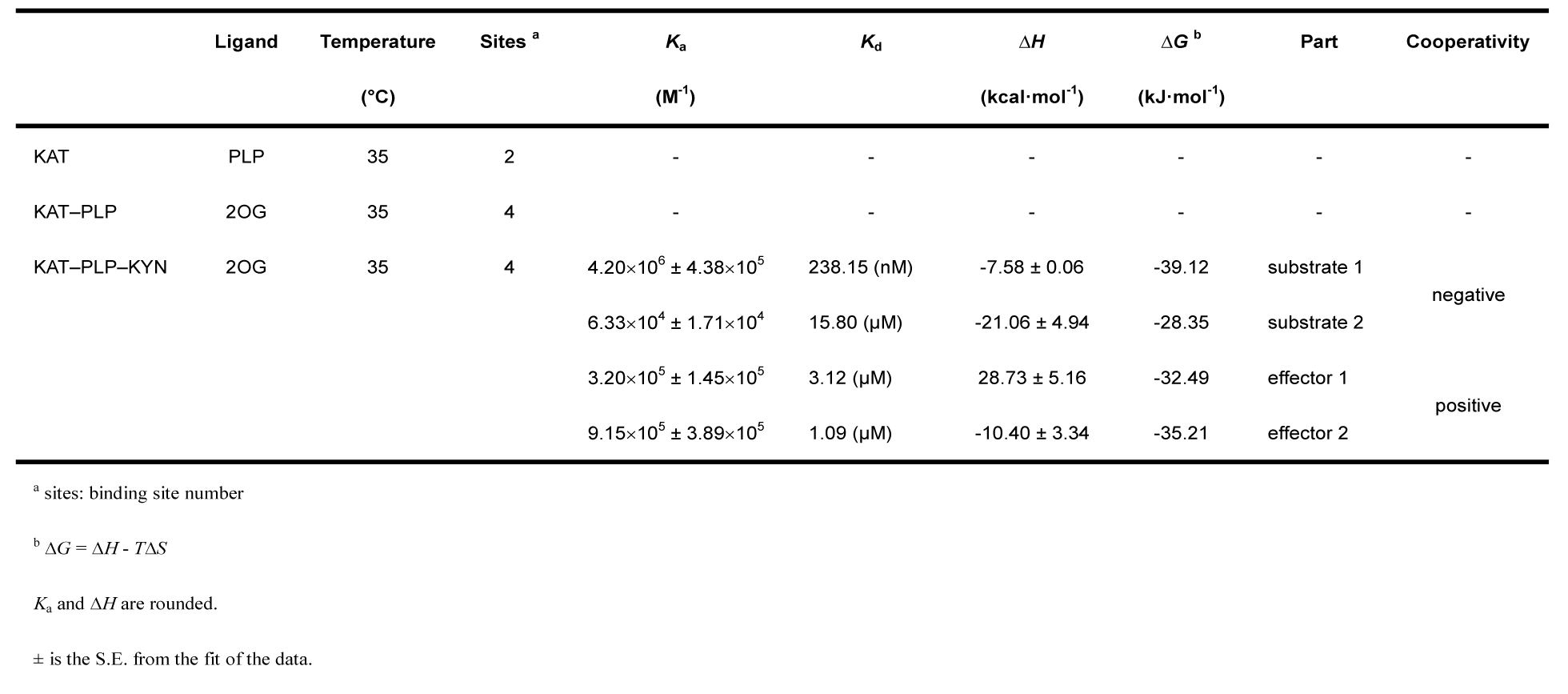

Supplement: Table S3 — ITC parameters of cofactor and substrate binding to PhKAT. All measurements were performed in 50 mM HEPES–NaOH buffer (pH 7.5) with 100 mM NaCl. (TIF) [file pone.0040307.s011.tif]

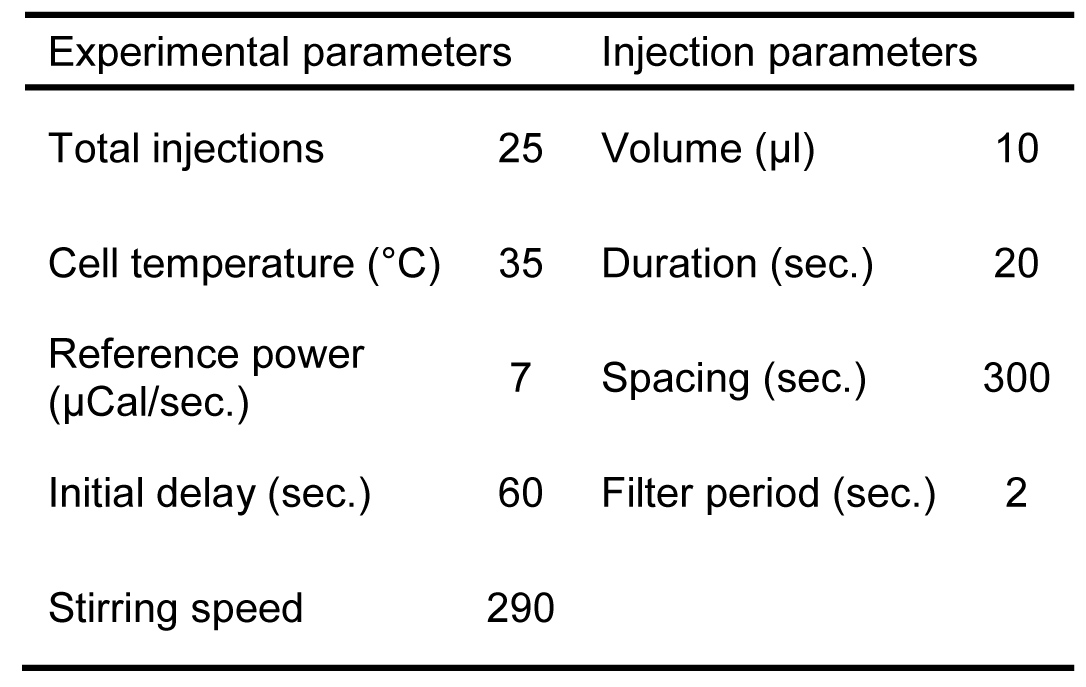

Supplement: Table S4 — ITC control parameters. (TIF) [file pone.0040307.s012.tif]

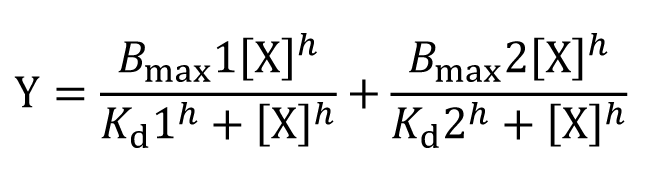

Supplement: Equation S1 — Two sites binding with hill slopes (altered two sites binding model). (TIF) [file pone.0040307.s013.tif]

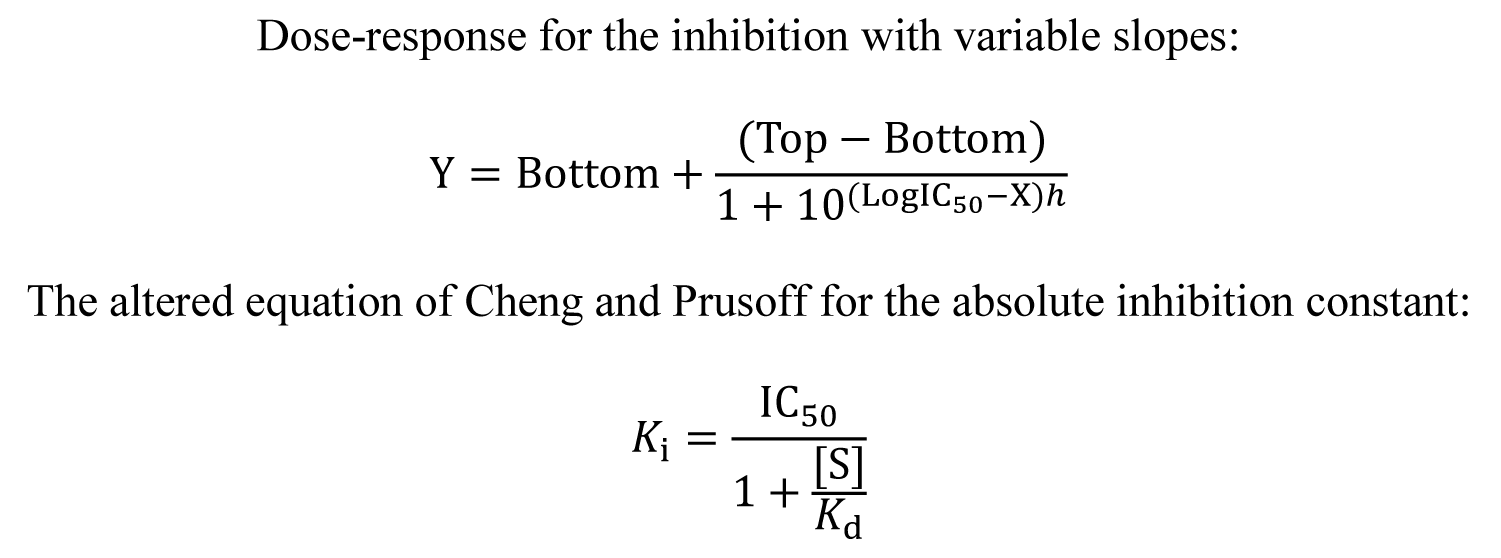

Supplement: Equation S2 — Sigmoidal dose-response and absolute inhibition constant ( K i). The IC50 value was converted to an absolute inhibition constant K i using the altered Cheng-Prusoff equation. (TIF) [file pone.0040307.s014.tif]

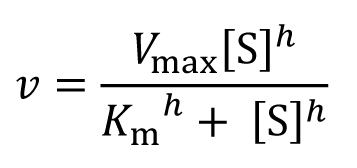

Supplement: Equation S3 — Allosteric sigmoidal. (TIF) [file pone.0040307.s015.tif]
